# Persistent oral health inequality in children—repeated cross-sectional studies in 2010 and 2019

**DOI:** 10.1186/s12889-024-20905-y

**Published:** 2024-12-18

**Authors:** Caroline Blomma, Thomas Davidson, Elisabeth Wärnberg Gerdin, Mats Bågesund, Johan Lyth

**Affiliations:** 1 Public Dental Service Östergötland, and Department of Health, Medicine and Caring Sciences, Linköping University, Linköping, Sweden; 2 Department of Health, Medicine and Caring Sciences, Linköping University, 581 83 Linköping, Sweden; 3 Centre for Orthodontics and Paediatric Dentistry, Norrköping, Department of Biomedical and Clinical Sciences, Linköping University, Linköping, Sweden

**Keywords:** Epidemiology, Caries, Socioeconomic status, Preschool children, Family, Residential area

## Abstract

**Background and aim:**

Children growing up in vulnerable circumstances have a higher risk of caries experience. Tracking the development of caries in relation to socioeconomic variables over time is essential for fair resource distribution to groups with higher caries risk and to even out inequalities in oral health. The aim was therefore to analyse the association between 6-year-olds´ caries prevalence and socioeconomic variables at family and residential area levels in 2010 and 2019 as well as potential differences in the association between 2010 and 2019.

**Methods:**

The study design is an epidemiological registry-based, repeated cross-sectional study based on caries data (grouped as 0, 1–3 and > 3 dmft) for the population of 6-year-olds in 2010 (*n* = 4,408, 95% coverage) and 2019 (*n* = 5,199, 94% coverage) in a Swedish region. Multiple socioeconomic variables for the children’s families and residential areas were retrieved from official registries. Multinomial logistic regression was performed at both levels to produce models for each level and studied year.

**Results:**

The variables that explained most of the association between caries and socioeconomic variables were mainly the same over the years at both levels. At the family level, these were: maternal age when having their first child (explained most of the association in both years, at 30 and 35%, respectively); maternal age when having the child in the study group; parental employment; parental and child’s migration background; maternal educational level; form of housing; and financial assistance (only 2010). At the residential area level, these were: migration background (explained most of the association both years; 82 and 52%, respectively), educational level and number of persons per household. The association between socioeconomic variables and caries was consistently stronger for severe caries (dmft > 3) than moderate (dmft 1–3). Multiple socioeconomic risk variables meant an even greater likelihood of caries.

**Conclusion:**

Over the studied years, variables related to socioeconomic vulnerability continued to be associated with caries in young children growing up under socially disadvantaged circumstances. Effective efforts for families living in socially vulnerable contexts are needed to achieve good and equal oral health, as is continued follow-up to evaluate whether the goal is reached.

**Supplementary Information:**

The online version contains supplementary material available at 10.1186/s12889-024-20905-y.

## Background

For decades it has been acknowledged that good health has become a particular benefit of more well-resourced individuals in society. First described by Black in England in the 1980s, this inequity in health has been established in studies worldwide [1–3]. Health inequity arises from inequity in the social conditions in which people are born, grow, work, live, and age [4]. Social determinants of health refer to an individual's living conditions, which are shaped by socio-economic and political contexts, in interaction with the individual's socioeconomic position such as income, education, occupation, and ethnicity, which also affects, for example, material circumstances, behaviours, and biological factors, ultimately affecting health outcomes. [5] In addition, there is a social gradient; health gradually deteriorates with a lower social position [4].

Inequalities in oral health follow the same pattern as inequalities in general health [6, 7]. Worldwide, 3.5 billion people suffer from poor oral health conditions and vulnerable groups are disproportionately affected [8]. Although largely preventable, dental caries remains one of the world's major non-communicable diseases (NCD) and untreated caries was the most common health condition globally in 2019, affecting one-third of the world’s population [9]. In total, 514 million children (1–9 years old) in the world are affected, and prevalence ranges from 46% in upper-middle-income countries to 38% in high-income countries [8]. In its severe form, caries can cause pain and adversely affect sleep, learning, and quality of life [10]. Caries in small children are related to continued caries progression later in life and poorer self-rated general health by midlife [11, 12].

Caries is preventable and if detected in its´ early stages also reversible [13]. However, preventive actions directed to the general public may increase oral health gaps as they mainly impact resourceful families and because families and children in socially vulnerable areas visit dental care to a lesser extent and are less likely to have access to, or take part in, caries prevention initiatives [14, 15]. Caries preventive actions directed toward at-risk children rather than to the entire population of children are also more cost-effective [16]. Family and area socioeconomic characteristics are suggested to identify families or areas with increased risk of disease to initiate preventive efforts or as a basis for the distribution of public resources [17–19]. For example, in regions in Sweden, a capitation per child patient is issued to dental care providers based on the socioeconomic conditions of the child’s residential area through the Care Need Index (CNI) [20].

In Sweden, the prevalence of caries has decreased over the last few decades and in an international comparison, oral health in Swedish children is fairly good [21]. Between 1973 and 2013, the proportion of caries-free preschool children (5-year-olds) increased from 9 to 69% [21]. However, in recent years, the positive trend regarding caries in children of preschool age has stagnated and the percentage of caries-free 6-year-olds decreased between the years 2010 and 2019 from 78 to 72%, and WHOS´s goal— of 80% of 6-years-old caries-free by 2020—was not achieved [22, 23]. Furthermore, despite the generally good oral health of the population and the fact that dental health services, including preventive measures, are free of charge for children and young people until the age of 23, inequalities in oral health due to unequal living conditions are also found in Sweden [24, 25]. Similar to international studies, a recent study from Sweden showed multiple family socioeconomic status variables (ethnicity, wealth, parental education, and employment) were associated with dental caries experience in children [26, 27]. Moreover, regardless of the family's social situation, their residential area can be related to children's oral health through the transmission of norms and behaviours in the social interaction in the immediate surroundings; the so-called ‘neighbourhood effect’ [28, 29]. A study showed that children in Sweden living in the most deprived areas were five times more likely to experience caries than peers living in the most affluent areas [30].

In order to even out the modifiable differences in oral health and enable a fair prioritisation of resources based on the population's needs, it is important to follow the development of caries and caries risk over time from a socioeconomic perspective to identify oral health inequities and at-risk groups [31, 32]. As the positive development in the proportion of caries-free 6-year-olds has stagnated in Sweden since 2010, it is of value to analyse how the association between family and residential area socioeconomic variables and caries prevalence in 6-year-olds has developed in recent years.

The overall aim of the study was to analyse the association between 6-year-olds´ caries prevalence and socioeconomic variables at family and residential area levels in 2010 and 2019 as well as potential differences in the association between 2010 and 2019.

## Methods

The study design is an epidemiological registry-based repeated cross-sectional study. It took place in the region of Östergötland, in southeast Sweden, which is the fourth largest region in Sweden with a population of 468,000 inhabitants (in year 2021) living in both rural and urban areas [33]. The dental health development in the region in terms of caries-free children follows national development [23, 34]. Basic oral health information is delivered to all children staying in the region, from pre-birth until 19 years of age, and their parents, with the aim of ensuring good opportunities for the children to maintain or achieve good oral health. Since 2006, resources have also been allocated for extended preventive efforts to be carried out in areas with low socioeconomic status based on area-based socioeconomics to reduce oral health differences within the region. As a part of the regional and national follow-up of oral health, dental health service providers (public and private dental clinics) in the region report epidemiological caries data to Region Östergötland for all examined 3, 6, 12 and 19-year-olds, and, since 2019, all 23-year-olds.

### Study group

Study groups consisted of the population of children (born 2004 and 2013) who, as 6-year-olds, (during 2010 and 2019), visited dental care in the region of Östergötland for oral examination. The population of 6-year-olds in the region was 4,630 in 2010 and 5,506 in 2019 [34, 35]. Those 6-year-olds who did not have caries data, a link to their residential area, or completely lacked socioeconomic variables were excluded.

### Data collection

The caries data were retrieved from the epidemiological caries data reported to Region Östergötland by dental health providers in the region labelled with the individual´s social security number (a unique ten-digit code for all Swedish residents). Caries prevalence variable (dependent variable) was measured in dmft, referring to the number of decayed, missing, or filled primary teeth and categorised into three groups: no caries (dmft = 0), moderate caries experience (dmft = 1–3), and severe caries experience (dmft > 3) [36, 37]. In this context, caries refers to manifest (dentin) caries; that is, caries that have passed through the enamel and into the dentin [21]. Caries were diagnosed at oral examinations based on clinical and radiographic findings by the child’s ordinary dental caregiver.

Statistics Sweden´s (SCB, responsible for official statistics in Sweden) classification of Sweden's municipalities into smaller geographical areas, so-called ‘key code areas’ (NYKO), was used as a definition for residential area (henceforth referred to as ‘area’). NYKO is available in several sizes, from single properties to larger areas [38]. Smaller areas expose more of the socioeconomic disparities in society compared to larger areas, to obtain as granular data as possible while maintaining confidentiality, one of the smallest divisions, the NYKO4 division, was used in the present study [20, 30].

Socioeconomic variables (independent variables) originated from official registries provided by SCB were used. The link between the children´s caries prevalence, socioeconomic information, and area was established by SCB through the social security numbers. Before the data records were delivered to researchers, the social security numbers were replaced with serial numbers. Variables were divided into relevant categories inspired by previous research [20, 26, 30, 39]. A complete description of each variable and associated categories is presented in Appendix 1.

Family-level variables were retrieved for either the children in the study group and/or their parents. Background data (gender), ethnicity, migration background, family type, and number of children in the household were retrieved for each child. Number of persons per household, disposable income, financial assistance, housing allowance, form of housing and urban or rural area were retrieved for each parent with whom the child was registered. Migration background, age when the child in the study group was born, age when the first child was born, employment, and educational level were retrieved on both the mother and the father respectively. In cases where an adoptive mother or father existed, these replaced the biological parent. Missing values (see Appendix 2a) in the variables on the family level were replaced using multiple imputation [40].

At the area level, socioeconomic data for the total population living in each area were retrieved. This included the following variables: background data (age and gender), ethnicity, migration background, age (when the first child was born), employment, education, family type, number of children in household, household size, disposable income and high-income households, financial assistance, housing allowance, form of housing, urban/rural area, and CNI. Data consisted of proportions or averages of inhabitants for each categorised variable.

### Analyses and statistics

To describe 6-year-olds´ caries prevalence and socioeconomic variables at family (non-imputed data) and area levels in 2010 and 2019, descriptive statistics were reported on categorical (frequency and percentage) and continuous variables (mean, standard deviation, and range) for both years. Chi^2^ tests (categorical variables) and independent sample t-tests (continuous variables) were performed to analyse potential differences between the studied years.

To analyse the association between 6-year-olds´ caries prevalence and socioeconomic variables, multinomial logistic regression models were performed at the family (imputed data) and area level for 2010 and 2019. The association between caries experience and socioeconomic variables was expressed by odds ratio (OR) and 95% confidence intervals (CI). To analyse potential differences in the associations between caries prevalence and socioeconomic variables, time interactions for each variable and caries category were tested. For significance testing on imputed data the median, pooling rules were used for *p*-values [41]. For the area level, NYKO codes were used as random intercepts to address potential correlations between persons living in the same area.

Primarily, univariable logistic regression analysis for each independent variable per studied level and year was performed. Secondly, four adjusted multivariable models were analysed; one for each level and each studied year (background variables excluded). Multicollinearity was tested using variance inflation factors (VIF), and values over five were considered problematic. If two independent variables were strongly correlated with each other according to VIF, the variable with the strongest association with 6-year-olds´ caries prevalence was included in the model.

At the family level, all variables were included in the models. At the area level, the included variables (and categories) were: migration background (proportion with foreign background), parental age when the first child was born (average age of mothers/fathers at the birth of their first child), employment (proportion without employment), educational level (proportion with highest attained education elementary school), family type (proportion of single mothers/fathers), number of persons/ household (average number of persons/ household), disposable income (median disposable income/consumption unit/household divided by 100,000), financial assistance (proportion of households receiving financial assistance), housing allowance (proportion of households with housing allowance), form of housing (proportion of families renting), urban/rural area (proportion of households in urban areas), and area CNI (> 1). Ethnicity, number of children/ household and proportion of high-income households were excluded due to multicollinearity.

Descriptive figures were created to visualise changes in caries prevalence between the studied years and different groups based on relevant socioeconomic variables and relevant cut-offs for each variable. Absolute (difference in proportion of caries-free children) and relative (unadjusted OR) inequalities were calculated.

Goodness of fit (pseudo-R2) for multivariable models were assessed with McFadden and Nagelkerke. Statistical significance level was set to < 0.05. All analyses were performed using the Statistical Package for Social Sciences (SPSS vn.29) and R v.4.2.3.

## Results

### Population characteristics

In 2010 4,431 6-year-olds (96%) had an oral examination of which 4,408 (95%) were included in the study as they met the inclusion criteria with eligible data for caries, area and socioeconomic variables. Corresponding figures in 2019 were 5,206 examined 6-year-olds (95%) of which 5,199 (94%) could be included in the study. In total 222 6-year-olds were excluded in 2010 and 307 in 2019. The population was distributed within 622 areas in 2010 and 651 in 2019.

On the family level, there was a difference (*p* = < 0.001) in caries experience between the studied years. In 2010, 78% of the children in the study group were caries-free (dmft = 0); for 2019, the corresponding proportion was 74% and the proportion with dmft > 3 increased from 9.3% to 11.5% (*p* < 0.001). The percentile with the most caries had dmft of 3 or above in 2010 and dmft 4 or above in 2019; for the other percentiles the dmft remained unchanged (not included in the table). The mean dmft among 6-year-olds per area was 0.74 in 2010 and 0.85 in 2019 (*p* = 0.148).

### Descriptive data for caries prevalence and socioeconomic variables

Tables 1 and 2 presents the main results for descriptive data on caries prevalence and the distribution of socioeconomic variables at family and area levels for 2010 and 2019 and whether there were significant differences in proportions between the studied years. For the full table, see Appendix 2a–b. At the family level, all variables but four displayed significant differences in proportions between 2010 and 2019 (Table 1), e.g., the proportions of children born in Sweden had decreased from 94 to 93%, and the proportion of children born outside Europe grew by 1.3 percentage points (pp). The proportions of children and parents with foreign migration backgrounds had increased among children from 17 to 23% and for parents from 22 to 30%. For maternal age when the child in study group, and for when the first child was born, there was an increase in proportions of parents > 35 years of age from 19 to 22%, from 6.3% to 7.5%, respectively; and a decrease of proportions of mothers aged 25–34y from 67 to 63% from 62 to 61%. The proportion of mothers with the highest attained education being high school had decreased from 43 to 38%, while the proportion with only elementary school (low educational level) had increased from 10 to 14%.

**Table 1:** Main results for descriptive data on caries and socioeconomic variables 2010 and 2019 (family level)

**Variable**		**2010**	**2019**	
	Category	n (%)	n (%)	*p*-value
**6y**		4,408	5,199	
**Caries (dmft)**	0	3,426 (77.7)	3,838 (73.8)	
	1–3	574 (13.0)	764 (14.7)	
	> 3	408 (9.3)	597 (11.5)	< 0.001
**Childs gender**	Male	2,260 (51.3)	2,691 (51.8)	
	Female	2,148 (48.7)	2,508 (48.2)	0.632
**Childs´ ethnicity**	Sweden	4,156 (94.3)	4,813 (92.6)	
	Europe except Sweden	54(1.2)	84 (1.6)	
	Outside of Europe	198 (4.5)	301 (5.8)	< 0.01
**Childs´ migration background**	Native^1^	3,678 (83.4)	3,998 (76.9)	
	Foreign^2^	730 (16.6)	1201 (23.1)	< 0.001
**Maternal migration background**	Native	3,438 (78.2)	3,617 (69.7)	
	Foreign	959 (21.8)	1,573 (30.3)	< 0.001
**Paternal migration background**	Native	3,394 (78.3)	3,541 (69.7)	
	Foreign	942 (21.7)	1,537 (30.3)	< 0.001
**Maternal age when child in the study group was born**	< 20y	76 (1.7)	64 (1.2)	
	20-24y	530 (12.0)	686 (13.2)	
	25-34y	2,970 (67.4)	3,291 (63.4)	
	> 35y	829 (18.8)	1,152 (22.2)	< 0.001
**Paternal age when child in the study group was born**	< 20y	29 (0.7)	19 (0.4)	
	20-24y	208 (4.8)	326 (6.4)	
	25-34y	2,604 (59.6)	2,770 (54.4)	
	> 35y	1,528 (35.0)	1,977 (38.8)	< 0.001
**Maternal age when first child was born**	< 20y	295 (6.7)	300 (5.8)	
	20-24y	1,100 (25.0)	1,356 (26.1)	
	25-34y	2,726 (62.0)	3,145 (60.6)	
	> 35y	276 (6.3)	389 (7.5)	< 0.01
**Paternal age when their first child was born**	< 20y	70 (1.6)	85 (1.7)	
	20-24y	630 (14.5)	749 (14.7)	
	25-34y	2,953 (68.1)	3,359 (66.1)	0.144
**Maternal employment status**	Employed	3,475 (79.0)	4,221 (81.4)	
	Unemployed	922 (21.0)	965 (18.6)	< 0.01
**Maternal educational level**	Elementary school	446 (10.3)	601 (13.7)	
	Highschool	1,889 (43.4)	1,676 (38.2)	
	Higher education	2,015 (46.3)	2,110 (48.1)	< 0.001
**Paternal educational level**	Elementary school	460 (10.7)	637 (14.2)	
	Highschool	2,186 (50.7)	2,116 (47.3)	
	Higher education	1,669 (38.7)	1,722 (38.5)	< 0.001
**Family type**	Single parent	738 (16.7)	968 (18.6)	
	Not single	3,668 (83.3)	4,226 (81.4)	0.016
	Else	2	5	
**Number of children in the household**	1	465 (10.6)	609 (11.7)	
	2–3	3,499 (79.4)	3,938 (75.8)	
	≥ 4	442 (10.0)	647 (12.5)	< 0.001
**Number of persons/ household**	1–5	3,997 (91.1)	3,999 (98.7)	
	> 5	392 (8.9)	52 (1.3)	< 0.001
**Familys´ disposable income**	Median	506,770	572,050	< 0.001
**Financial** **assistance**	Yes	385 (8.8)	176 (5.9)	
	No	4,004 (91.2)	5,002 (96.6)	< 0.001
**Housing allowance**	No	3,656 (82.9)	4,246 (82.0)	
	Yes	733 (16.7)	932 (18.0)	0.095
**Form of housing**	Renting	1,186(26.6)	965(23.8)	
	Owning appartment	159(3.6)	186(4.6)	
	Owning house	3,022(68.9)	2,840(70.2)	< 0.01
	Else	40	56	
**Family living in in urban or rural**	Urban	3,707 (84.1)	4,481 (86.2)	
	Rural	701 (15.9)	718 (13.8)	0.004

^a^native = Born in Sweden with one or two native Swedish parents, ^b^foreign = Born abroad or born in Sweden with two foreign born parents

Results for descriptive data on caries prevalence and socioeconomic variables at family level, for year 2010 and 2019 and significant differences in proportions between the years

**Table 2:** Main results on descriptive data for caries and socioeconomic variables 2010 and 2019 (area level)

**Variable**		**2010**	**2019**	
	Category	n (%)	n (%)	*p*-value
**NYKO**		622	651	
**N 6y /NYKO**	Mean (SD)	7.1 (8.4)	8.0 (9.9)	0.08
	Range	1- 64	1- 71	
**Population**	Mean (SD)	658 (703)	685 (746)	0.51
	Range	11–4,298	4–4,932	
**Caries** **(Mean dmft/ NYKO)**	Mean (SD)	0.74 (1.31)	0.85 (1.35)	0.15
	Range	0.0- 10.0	0.0- 10.5	
**Proportion of male**	Mean (SD)	50.7 (3.4)	51. 1 (3.1)	0.04
	Range	36.0–72.7	37.5–72.7	
**Proportion born in Sweden**	Mean (SD)	91.3 (8.6)	88.3 (11.3)	< 0.001
	Range	32.4–100.0	20.0–100.0	
**Proportion with foreign migration background**	Mean (SD)	11.2 (11.2)	15.3 (14.9)	< 0.001
	Range	0.0- 79.4	0.0–100.0	
**Average age of mothers at the birth of their first child**	Mean (SD)	27.6 (3.7)	28.4 (3.4)	< 0.001
	Range	17–41	19–41	
**Average age of fathers at the birth of their first child**	Mean (SD)	30.4 (3.8)	30.4 (3.9)	0.965
	Range	20–51	20–51	
**Proportion with employment**	Mean (SD)	50.2 (7.1)	51.0 (7.6)	0.045
	Range	21.3–73.4	0.0–73.5	
**Proportion with highest education elementary school**	Mean (SD)	14.5 (6.6)	10.8 (6.7)	< 0.001
	Range	0.0–39.0	0.0–42.1	
**Proportion with highest education high school**	Mean (SD)	50.3 (10.3)	48.4 (11.5)	< 0.001
	Range	8.3–73.3	0.0–81.8	
**Proportion with higher education**	Mean (SD)	33.7 (14.1)	38.9 (14.5)	< 0.001
	Range	5.4–91.7	0.0–100.0	
**Proportion of single mothers**	Mean (SD)	4.0 (3.6)	3.6 (3.0)	0.045
	Range	0.0–40.0	0.0–25.0	
**Proportion of single fathers**	Mean (SD)	1.5 (1.7)	1.7 (1.6)	0.066
	Range	0.0–20.0	0.0–16.7	
**Average number of children/ household**	Mean (SD)	0.69 (0.28)	0.66 (0.29)	0.03
	Range	0.04–1.75	0.04–2.00	
**Average number of persons/ household**	Mean (SD)	2.4 (0.43)	2.3 (0.43)	0.07
	Range	1.3–3.8	1.3–4.0	
**Median disposable income per consumption unit/ household**	Mean (SD)	233,007 (38,829)	450,842 (135,465)	< 0.001
	Range	104,424–393,913	134,768–1,123,667	
**Proportion of high-income households**	Mean (SD)	10.8 (4.3)	11.1 (4.4)	0.1
	Range	0.0–30.8	0.0–27.3	
**Proportion of households with financial assistance**	Mean (SD)	3.6 (5.7)	2.4 (4.6)	< 0.001
	Range	0.0–43.5	0.0–30.6	
**Proportions housing renting**	Mean (SD)	21.7 (30.1)	21.9 (30.1)	0.899
	Range	0–100	0–100	
**Proportion owning appartment in appartment building**	Mean (SD)	6.5 (17.0)	7.6 (17.9)	0.285
	Range	0–100	0–100	
**Proportion owning house**	Mean (SD)	66.0 (36.7)	66.2 (38.)	0.91
	Range	0–100	0–100	
	Std.Dev	36.7	38.1	
**Other type of housing**	Mean	5.8	4.3	
**Proportion of households in urban area**	Mean	65.7	66.3	0.824
	Range	0–100	0–100	
	Std.Dev	46.1	45.8	
	Missing	-	1	
**Residential area CNI**	Mean	1.14	1.19	0.148
	Range	0.3–4.0	0.2–6.3	
	Std.Dev	0.5	0.7	

Main results for descriptive data on caries prevalence and social and socioeconomic variables at residential area level for year 2010 and 2019 and significant differences in proportions between the years

In Table 2, the main results for caries prevalence and socioeconomic variables at the area level for 2010 and 2019 are presented. Background data (age, gender), ethnicity, migration background, maternal age (when the first child was born), employment, education, family type, number of children per household, and disposable income displayed significant differences in proportions between studied years; E.g., the mean proportion born in Sweden had decreased from 91 to 88% and the mean proportion with foreign migration background had increased from 11 to 15%. The mean average maternal age when the first child was born had increased from 27.6 years to 28.4 years. The mean proportion of parents with higher education had increased from 34 to 39%, while the mean proportion with elementary or high-school education had decreased from 15 to 11% and from 50 to 48%, respectively. The mean average number of persons per household was 2.4 in 2010 and 2.3 in 2019 (*p* = 0.066).

### Association between caries prevalence and socioeconomic variables

#### Family level

In the univariable regression analysis at the family level, all socioeconomic variables included in the study were associated with the likelihood of caries prevalence in both years (except gender and number of persons/ households in 2019). The association between socioeconomic variables and caries prevalence was consistently stronger for severe caries than for moderate. For the variables maternal age when the child in the study group was born, parental age when the first child was born, maternal educational level, family type, number of persons per household, financial assistance and form of housing OR were significantly lower in 2019 than in 2010. A selection of socioeconomic variables and their association with caries on the family level in 2010 and 2019 are presented in Table 3 (for the full table, see Appendix). Main results of the univariable analysis on the association between caries and a selection of socioeconomic variables on family level year 2010 respectively 2019 and differences in OR between the studied years (p-interaction)

**Table 3:** Main results of univariable analysis of the association between caries and socioeconomic variables (family level)

**Variable (ref category)**	Year	Category	**Comparison dmft 1–3 against 0**					**Comparison dmft > 3 against 0**				
			***P*-value**	**OR**	**CI Lower**	**CI Upper**	***p*-interaction**	***p*-value**	**OR**	**CI Lower**	**CI Upper**	**p-interaction**
**Child´s ethnicity (native)**	2010	Europe	0.16	1.76	0.80	3.88		< 0.001	6.18	3.36	11.37	
		Outside Europe	< 0.001	3.87	2.65	5.64		< 0.001	10.53	7.52	14.79	
	2019	Europe	0.76	1.12	0.54	2.31	0.71	< 0.001	6.45	4.04	10.30	0.68
		Outside Europe	< 0.001	3.72	2.69	5.14		< 0.001	12.77	9.66	16.89	
**Child´s migration background (native)**	2010	Foreign	< 0.001	3.96	3.20	4.92		< 0.001	16.01	12.71	20.17	
	2019	Foreign	< 0.001	3.39	2.84	4.03	0.27	< 0.001	14.12	11.60	17.18	0.42
**Maternal migration background (native)**	2010	Foreign	< 0.001	3.08	2.53	3.76		< 0.001	11.87	9.45	14.92	
	2019	Foreign	< 0.001	3.05	2.59	3.59	0.96	< 0.001	11.73	9.59	14.36	0.94
**Paternal migration background (native)**	2010	Foreign	< 0.001	3.16	2.60	3.85		< 0.001	10.47	8.31	13.19	
	2019	Foreign	< 0.001	2.83	2.40	3.33	0.37	< 0.001	11.07	9.04	13.56	0.76
**Maternal age when child in the study group was born (25-34y)**	2010	< 20y	< 0.01	2.62	1.40	4.92		< 0.001	9.17	5.44	15.48	
		20-24y	< 0.001	1.71	1.32	2.22		< 0.001	3.45	2.64	4.50	
		> 35y	0.21	1.16	0.92	1.46		< 0.01	1.48	1.13	1.95	
	2019	< 20y	< 0.01	2.49	1.28	4.87	0.66	< 0.001	6.32	3.62	11.03	< 0.01
		20-24y	< 0.01	1.44	1.15	1.81		< 0.001	2.12	1.69	2.66	
		> 35y	0.96	1.00	0.82	1.21		0.06	0.80	0.63	1.01	
**Maternal age when first child was born (25-34y)**	2010	< 20y	< 0.001	3.15	2.26	4.39		< 0.001	12.53	9.15	17.17	
		20-24y	< 0.001	2.17	1.78	2.65		< 0.001	4.04	3.15	5.18	
		> 35y	0.50	0.86	0.56	1.33		0.12	1.49	0.90	2.46	
	2019	< 20y	< 0.001	2.96	2.17	4.05	0.83	< 0.001	7.74	5.76	10.41	0.03
		20-24y	< 0.001	1.92	1.61	2.28		< 0.001	3.65	3.00	4.44	
		> 35y	0.32	0.85	0.61	1.18		0.09	0.65	0.39	1.07	
**Maternal employment status (Yes)**	2010	No	< 0.001	2.83	2.32	3.45		< 0.001	8.65	6.94	10.77	
	2019	No	< 0.001	2.80	2.32	3.36	0.94	< 0.001	6.82	5.65	8.23	0.11
**Paternal employment status (Yes)**	2010	No	< 0.001	2.46	1.91	3.17		< 0.001	8.41	6.61	10.69	
	2019	No	< 0.001	2.49	1.95	3.18	0.89	< 0.001	6.46	5.22	8.00	0.12
**Maternal level of education** **(Higher education)**	2010	Elementary school	< 0.001	3.94	2.98	5.22		< 0.001	14.99	11.15	20.16	
		Highschool	< 0.001	1.54	1.27	1.87		< 0.001	1.89	1.44	2.49	
	2019	Elementary school	< 0.001	2.94	2.30	3.76	0.11	< 0.001	8.01	5.34	12.03	< 0.001
		Highschool	< 0.001	1.65	1.35	2.02		< 0.001	2.08	1.60	2.72	
**Number of children in the household (2–3)**	2010	1	0.12	1.25	0.95	1.66		0.21	1.25	0.88	1.78	
		> 3	< 0.001	2.34	1.79	3.06	0.58	< 0.001	5.22	4.039	6.752	0.15
	2019	1	0.07	1.25	0.98	1.58		0.37	0.87	0.64	1.18	
		> 3	< 0.001	2.80	2.261	3.463		< 0.001	4.06	3.28	5.04	
**Number of persons/ household (1–5)**	2010	> 5	< 0.001	2.10	1.58	2.79		< 0.001	4.82	3.70	6.27	
	2019	> 5	0.26	2.03	0.52	8.04	0.06	0.12	4.08	0.60	28.15	0.01
**Family’s disposable income** **(> highest quartile)**	2010	< lowest quintile	< 0.001	2.52	1.93	3.30		< 0.001	11.76	7.85	17.64	
		In between	< 0.01	1.45	1.15	1.83		< 0.001	3.26	2.19	4.86	
	2019	< lowest quintile	< 0.001	2.76	2.18	3.49	0.85	< 0.001	8.74	6.34	12.05	0.15
		In between	< 0.001	1.47	1.20	1.81		< 0.001	3.22	2.36	4.37	
**Financial assistance (No)**	2010	Yes	< 0.001	4.15	3.11	5.54		< 0.001	16.39	12.61	21.29	
	2019	Yes	< 0.001	4.10	2.77	6.06	0.91	< 0.001	8.34	5.83	11.91	< 0.01
**Form of housing (Owning house)**	2010	Renting	< 0.001	2.66	2.20	3.23		< 0.001	9.56	7.54	12.12	
		Owning apartment	0.11	1.48	0.92	2.38		< 0.001	2.89	1.63	5.13	
	2019	Renting	< 0.001	2.61	2.11	3.23		< 0.001	6.37	4.14	9.80	0.01
		Owning apartment	< 0.01	2.13	1.34	3.37	0.22	0.11	1.61	0.89	2.90	

Main results of the univariable analysis on the association between caries and a selection of socioeconomic variables on family level year 2010 respectively 2019 and differences in OR between the studied years (p-interaction).

The result of the multivariable models of the socioeconomic variables that explained most of the association with caries are presented in Table 4, arranged by the variable´s contribution of explanation to the 2019 model. The goodness of fit statistics were: McFadden = 0.17 (2010), 0.13 (2019); Nagelkerke = 0.28 (2010), 0.22 (2019). The variables that explained most of the association both years were maternal age upon having their first child, parental and child migration background, form of housing, paternal employment, maternal education, maternal age when the child in the study group was born, and financial assistance—but with some variation in the proportion of explanation between the years. Financial assistance was included in the 2010 model but not in the 2019´s. For maternal educational level OR for caries experience was significantly lower (*p* ≤ 0.01) in 2019 than in 2010 (e.g. for low educational level OR 14.99 y2010, OR 8.01 y2019 for severe caries). Very young/young maternal age when the first child was born explained most of the association (35% in 2010 and 30% in 2019) in both years. Both groups of children whose mothers had their first child before the age of 25: < 20yrs and 20-24yrs; were more than twice as likely to suffer severe caries disease than children whose mothers were between 25 and 34 years when they had their first child, in 2010 as well as in 2019. Children with multiple risk variables had an even higher likelihood of caries experience. For instance, in 2019 the OR for severe caries for children of maternal age when the first child was born < 20 years and with a foreign maternal background was 6.0 (2.6*2.3).

**Table 4:** The multivariable models for 2010 and 2019´s association between caries and socioeconomic variables (family level)

					**2010**							
					**Comparison 1-3 against 0**				**Comparison >3 against 0**			
**Variable**	**Category**	**Degree of freedom**	χ^2^ **values (Likelihood ratio)**	**Model explanation** **(%)**	***P-*value**	**OR**	**CI Lower**	**CI Upper**	***p-*value**	**OR**	**CI Lower**	**CI Upper**
**Maternal age when first child was born**		7	62.45	34.9								
	**<20y**				<0.01	2.00	1.31	3.04	<0.001	4.20	2.69	6.57
	**20-24y**				<0.001	1.72	1.34	2.20	<0.001	2.09	1.50	2.90
	**25-34y**					1				1		
	**>35y**				0.07	0.64	0.39	1.04	0.64	0.86	0.47	1.60
**Maternal migration background**		3	9.51	5.3								
	**Foreign**				0.23	1.23	0.87	1.74	<0.01	2.03	1.30	3.17
	**Native**					1				1		
		5	11.29	6.3								
**Form of housing**	**Renting**				0.10	1.23	0.96	1.58	<0.01	1.60	1.15	2.22
	**Owning apartment **				0.40	1.24	0.76	2.02	0.05	1.88	1.01	3.48
	**Owning house**					1				1		
**Paternal employment status **		3	2.41	1.3								
	**No**				0.59	0.92	0.67	1.26	0.25	1.22	0.87	1.72
	**Yes**					1				1		
**Maternal level of education**		5	35.69	20.0								
	**Elementary**				<0.01	1.71	1.22	2.39	<0.001	2.85	1.94	4.17
	**Highschool**				0.01	1.31	1.06	1.61	0.01	1.47	1.08	2.00
	**Higher education**					1				1		
**Childs´ migration background **		3	18.17	10.2								
	**Foreign**				0.01	1.74	1.14	2.66	<0.001	2.83	1.65	4.83
	**Native**					1				1		
**Maternal employment**		3	14.26	8.0								
	**No**				<0.01	1.44	1.12	1.85	<0.01	1.64	1.20	2.23
	**Yes**					1				1		
**Maternal age when child in the study group was born**		7	14.55	8.1								
	**<20y**				0.11	0.77	0.56	1.06	0.36	0.71	0.34	1.47
	**20-24y**				0.51	0.78	0.37	1.65	0.58	0.90	0.63	1.30
	**25-34y**					1				1		
	**>35y**				0.04	1.33	1.02	1.75	<0.01	1.63	1.15	2.33
**Paternal migration background**		3	3.50	2.0								
	**Foreign**				0.08	1.34	0.97	1.86	0.37	1.24	0.77	2.00
	**Native**					1				1		
**Financial ** **assistance **		3										
	**Yes**		6.87	3.8	0.23	1.27	0.86	1.86	0.01	1.65	1.12	2.42
	**No**					1				1		

					2019							
					Comparison 1-3 against 0				Comparison >3 against 0			
Variable	**Category**	**Degree of freedom**	χ^2^ values (Likelihood ratio)	Model explanation (%)	*p*-value	OR	CI Lower	CI Upper	*p*-value	OR	CI Lower	CI Upper
Maternal age when first child was born		7	52.99	29.5								
	**<20y**				0.01	1.70	1.11	2.58	<0.001	2.59	1.63	4.14
	**20-24y**				<0.01	1.49	1.18	1.89	<0.001	2.02	1.51	2.70
	**25-34y**					1			1			
	**>35y**				0.07	0.69	0.47	1.03	0.01	0.44	0.23	0.83
Maternal migration background		3	23.37	13.0								
	**Foreign**				<0.001	1.66	1.25	2.20	<0.001	2.25	1.50	3.36
	**Native**					1				1		
		5	21.42	11.9								
Form of housing	**Renting**				0.01	1.40	1.09	1.80	0.03	1.38	1.03	1.85
	**Owning apartment **				0.04	1.65	1.04	2.63	0.43	0.78	0.42	1.46
	**Owning house**					1				1		
Paternal employment status		3	20.76	11.5								
	**No**				0.07	1.31	0.98	1.77	<0.001	1.99	1.49	2.66
	**Yes**					1				1		
Maternal level of education		5	16.31	9.1								
	**Elementary**				0.12	1.36	0.92	2.01	0.02	1.63	1.07	2.49
	**Highschool**				0.02	1.38	1.07	1.78	0.03	1.49	1.05	2.14
	**Higher education**					1				1		
Childs´ migration background		3	15.66	8.7								
	**Foreign**				0.27	1.23	0.85	1.78	<0.001	2.39	1.53	3.73
	**Native**					1				1		
Maternal employment		3	12.60	7.0								
	**No**				0.01	1.34	1.07	1.69	<0.01	1.50	1.16	1.95
	**Yes**					1				1		
Maternal age when child in the study group was born		7	9.42	5.2								
	**<20y**				0.88	0.94	0.39	2.23	0.27	1.58	0.70	3.55
	**20-24y**				0.08	0.76	0.57	1.03	0.51	0.89	0.64	1.25
	**25-34y**					1				1		
	**>35y**				0.11	1.21	0.96	1.53	0.25	1.20	0.88	1.66
Paternal migration background		3	7.27	4.0								
	**Foreign**				0.20	1.21	0.91	1.61	0.01	1.72	1.14	2.59
	**Native**					1				1		
Financial assistance		3	0.08	0.0								
	**Yes**				0.61	1.16	0.65	2.06	0.80	1.06	0.66	1.72
	**No**					1				1		

**Fig. 1:**
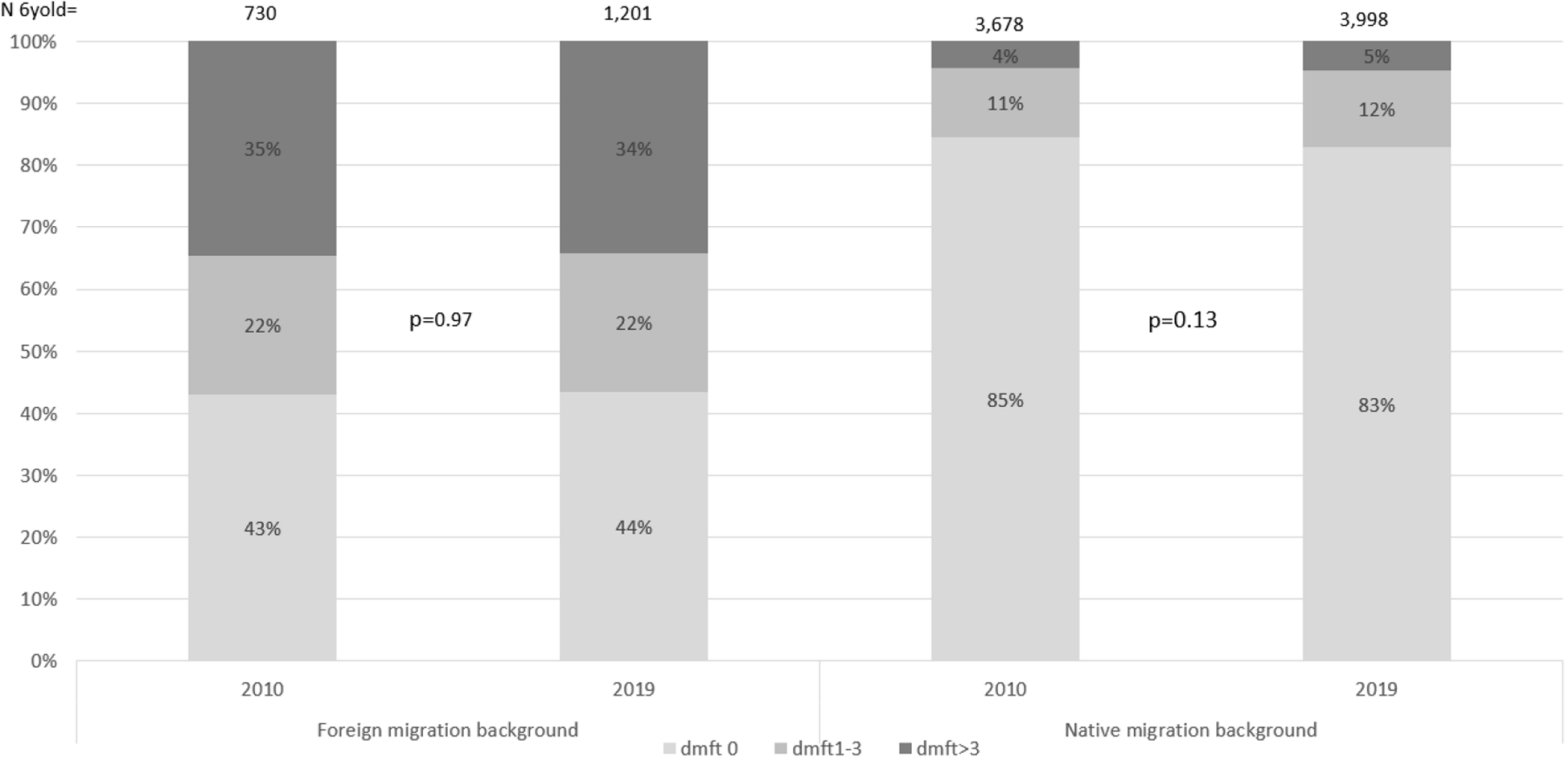
Child´s migration background and caries prevalence 2010 and 2019 at family level

The proportion of children and parents with foreign migration background had increased between 2010 and 2019 (Table 1), and parental and child migration background remained one of the most important explanatory variables in the models, explaining the association between caries and socioeconomic variables in 2010 and 2019 (Table 4). Figure 1 presents a comparison of the caries prevalence between 2010 and 2019 for children with foreign migration background and native migration background. The proportion of caries-free children with foreign migration background was unchanged between 2010 and 2019 (*p* = 0.97), the proportion of caries-free children with native migration background was 2pp lower in 2019 than in 2010 (*p* = 0.13). The unadjusted OR for caries prevalence (dmft > 0) for children with foreign migration backgrounds in relation to children with native migration backgrounds was 7.3 in 2010 and 6.7 in 2019.

**Fig. 2:**
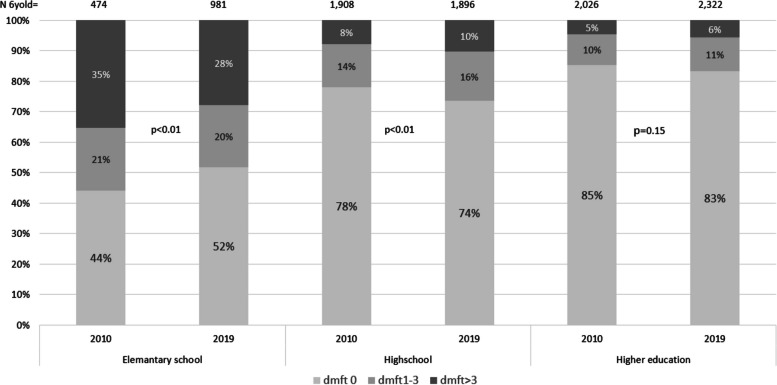
Maternal level of education and caries prevalence in 2010 and 2019 on family level

In the models (Table 4), OR for caries prevalence for children with low maternal level of education was significantly lower in 2019 than in 2010 in comparison to higher levels of education. Figure 2 presents the caries prevalence in relation to maternal level of education in 2010 respective to 2019 at the family level. The proportion of caries-free children with low maternal levels of education had increased from 44% in 2010 to 52% in 2019 (*p* < 0.01). The proportion of caries-free children with higher maternal levels of education was 2pp lower in 2019 (83%) compared to 2010 (85%) (*p* = 0.15). Hence, the difference in the proportion of caries-free children between children with the lowest and highest maternal level of education was 10pp lower in 2019 than in 2010, from 41 (85%−44%) to 31 percentage points (83%−52%). The unadjusted OR for children with low maternal education, in relation to children with high maternal education, was 7.38 in 2010 and decreased to 4.62 in 2019 (*p* < 0.001).

#### Area level

In the univariable analysis at area level, all socioeconomic variables included in the study showed associations with the likelihood of caries prevalence in both 2010 and 2019 and the associations were stronger for severe caries than moderate (Table 5). However, for ethnicity or migration background (foreign), employment (proportion of unemployed), level of education (proportion with elementary or high-school education), family type (proportion of single mothers), (proportions of households with) financial assistance, or housing allowance, the OR for severe caries was lower in 2019 than in 2010. For paternal age when the first child was born, (median) disposable income per consumption unit/ household, (proportion) of high-income households, the OR was less than 1 in both years and had increased. Children growing up in areas with CNI > 1 were significantly more likely (OR 5.98 y2010, OR 4.72 y2019) to suffer severe caries compared to children growing up in areas with CNI < 1. The full table can be found in Appendix 4.

**Table 5:** Main results of univariable analysis of the association between caries and socioeconomic variables (area level)

**Variable** **(Reference)**	Year	Category	**Comparison 1–3 against 0**					**Comparison > 3 against 0**				
			***p*-value**	**OR**	**CI Lower**	**CI Upper**	**p-interaction**	***p*-value**	**OR**	**CI Lower**	**CI Upper**	**p-interaction**
**Ethnicity (Proportion born in Sweden)**	**2010**	**Proportion born outside Sweden**	< 0.001	1.04	1.04	1.05		< 0.001	1.09	1.08	1.10	
	**2019**		< 0.001	1.03	1.03	1.04	0.02	< 0.001	1.06	1.05	1.06	< 0.001
**Migration background (Proportion with native migration background)**	**2010**	**Proportion with foreign background**^4^	< 0.001	1.03	1.03	1.04		< 0.001	1.07	1.06	1.07	
	**2019**		< 0.001	1.02	1.02	1.03	0.03	< 0.001	1.04	1.04	1.04	< 0.001
**Maternal age when first child was born**	**2010**	**Average age of mothers at the birth of their first child**	< 0.001	0.90	0.87	0.92		< 0.001	0.80	0.77	0.83	
	**2019**		< 0.001	0.94	0.92	0.97	0.02	< 0.001	0.82	0.79	0.85	0.350
**Paternal age when their first child was born**	**2010**	**Average age of fathers at the birth of their first child**	< 0.01	0.96	0.93	0.984		< 0.001	0.86	0.83	0.90	
	**2019**		0.76	1.00	0.97	1.02	0.03	< 0.01	0.96	0.93	0.98	< 0.001
**Employment status**	**2010**	**Proportion without employment**	< 0.001	1.06	1.05	1.07		< 0.001	1.12	1.10	1.13	
	**2019**		< 0.001	1.04	1.03	1.05	0.06	< 0.001	1.08	1.07	1.10	< 0.01
**Education level** **(Proportion with higher education)**	**2010**	**Proportion with highest education elementary school**	< 0.001	1.05	1.03	1.07		< 0.001	1.10	1.09	1.12	
		**Proportion with highest education high school**	0.19	1.01	1.00	1.02		< 0.001	0.97	0.96	0.99	
	**2019**	**Proportion with highest education elementary school**	< 0.001	1.04	1.03	1.05	0.25	< 0.001	1.07	1.06	1.08	< 0.01
		**Proportion with highest education high school**	0.80	1.00	0.99	1.01	0.21	0.56	1.00	0.99	1.01	< 0.001
		**Else**	< 0.001	1.08	1.05	1.12	0.41	< 0.001	1.14	1.10	1.17	0.46
**Family type** **(not single)**	**2010**	**Proportion of single mothers**	< 0.001	1.12	1.09	1.15		< 0.001	1.23	1.20	1.27	
	**2019**		< 0.001	1.10	1.07	1.12	0.64	< 0.001	1.17	1.15	1.20	< 0.01
**Family type** **(not single)**	**2010**	**Proportion of single fathers**	0.57	0.98	0.90	1.06		0.01	0.86	0.77	0.96	
	**2019**		0.01	0.91	0.84	0.98	0.22	< 0.001	0.77	0.71	0.84	0.14
**Number of persons/ household**	**2010**	**Average number of persons/ household**	< 0.001	0.55	0.45	0.68		< 0.001	0.41	0.32	0.52	
	**2019**		< 0.001	0.69	0.58	0.83	0.12	< 0.001	0.44	0.36	0.54	0.62
D**isposable income per consumption unit/ household**	**2010**	**Median disposable income per consumption unit/ household (divided by 10 < 0.001)**	< 0.001	0.32	0.25	0.39		< 0.001	0.07	0.05	0.09	
	**2019**		< 0.001	0.41	0.35	0.48	0.04	< 0.001	0.16	0.13	0.19	< 0.001
**Form of housing (Proportion owning house)**	**2010**	**Proportions renting**	< 0.001	1.01	1.01	1.02		< 0.001	1.03	1.03	1.04	
	2019		< 0.001	1.01	1.01	1.015	0.65	< 0.001	1.03	1.02	1.03	0.65
**Residential area CNI (< 1)**	**2010**	** > 1**	< 0.001	1.98	1.65	2.38		< 0.001	5.99	4.55	7.87	
	**2019**		< 0.001	1.88	1.60	2.21	0.68	< 0.001	4.72	3.79	5.86	0.18

OR = Odds Ratio, CI = 95% Confidence Interval

Main results of the univariable analysis on the association between caries and a selection of socioeconomic variables on family level year 2010 respectively 2019 and differences in OR between the studied years (p-interaction)

The results of the multivariable models the socioeconomic variables and the associated categories that explained most of the association with caries at the area level are presented in Table 6, arranged by the variables contribution of explanation to the 2019 model. The goodness of fit statistics were: McFadden = 0.10 (2010), 0.08 (2019); Nagelkerke = 0.17 (2010), 0.14 (2019). The variables that explained most of the association between caries and socioeconomic variables were the same in both 2010 and 2019, but with some variation in the proportion of model explanation. These were proportions with foreign background, proportions with low education, and average number of persons per household. Proportions with foreign background explained most of the association in both years (82% in 2010 and 52% in 2019). The OR for proportions with foreign backgrounds was significantly lower in 2019 than in 2010 (*p* < 0.001), and the OR for the proportion with low education was significantly higher in 2019 than in 2010 (*p* < 0.001). There was a positive correlation (R = 0.62, *p* < 0.001) between the average number of persons per household in the area and the proportion of residents owning their own house. In 2019, the OR for severe caries was 1.03 for the proportion with a foreign migrant background and the proportion with the highest level of education elementary school (low education), which means that the odds increase by 1.03 for every pp with an increase proportion of residents with a foreign migration background or low education in the area. For example, for children growing up in an area with an increased proportion of inhabitants of 10pp with low education OR for severe caries was 1.34 (1.03^10), compared to an area with a lower proportion. If the proportion of inhabitants with foreign migration background also was 10pp higher, then OR was 1.81 (1.03^10*1.03^10).

**Table 6:** The multivariable models for 2010 and 2019 association between caries and socioeconomic variables at area level

Variabel (category)	2010											2019									
		$${\mathbf{x}}^{\begin{array}{c}2\\ \end{array}}$$ **values**	**Model**	**Comparison dmft 1–3 against 0**				**Comparison dmft > 3 against 0**				$${\mathbf{x}}^{\begin{array}{c}2\\ \end{array}}$$ **Values**	**Model**	**Comparison dmft 1–3 against 0**				**Comparison dmft > 3ainst 0**			
	**Degrees of freedom**	**(Likelihood ratio)**	**explanation** **%**	***p*-** **value**	**OR**	**CI Lower**	**CI Upper**	***p*-** **value**	**OR**	**CI Lower**	**CI Upper**	**(Likelihood ratio)**	**explanation** **%**	**p-value**	**OR**	**CI Lower**	**CI Upper**	***p*-value**	**OR**	**CI Lower**	**CI Upper**
**Migration background** **(Proportion with foreign background)**	2	203.0	82	< 0.001	1.02	1.01	1.03	< 0.001	1.06	1.06	1.07	87.5	52	< 0.001	1.02	1.01	1.03	< 0.001	1.03	1.03	1.04
**Educational level** **(Proportion with highest education elementary school)**	2	27.8	11	< 0.001	1.04	1.02	1.06	< 0.001	1.02	1.01	1.02	68.7	41	0.28	1.01	0.99	1.03	< 0.001	1.03	1.03	1.03
**Number of persons per household** **(Average number of persons/ household)**	2	17.9	7	0.02	0.74	0.59	0.95	< 0.001	0.52	0.52	0.52	11.9	7	< 0.01	0.72	0.59	0.89	< 0.001	0.43	0.42	0.43

Abbreviations: OR = Odds ratio, CI = 95% Confidence intervals. Pseudo-R^2^: McFadden = 0.10 (2010), 0.08 (2019); Nagelkerke = 0.17 (2010), 0.14 (2019)

**Fig. 3:**
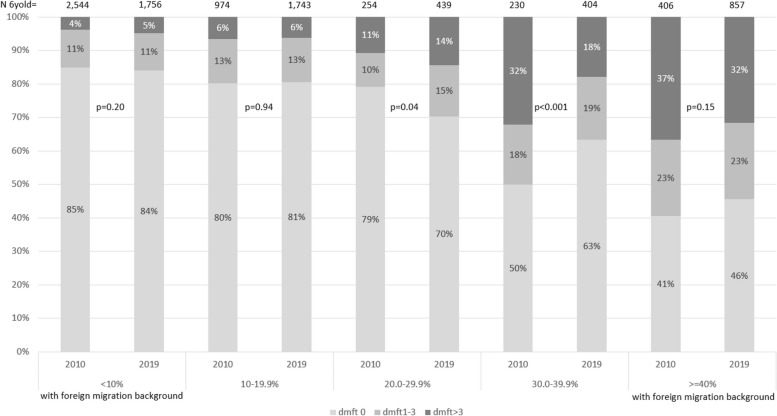
Caries prevalence by residential area based on the proportion of inhabitants with foreign background

There was a gradient of increased prevalence of caries for areas with an increased proportion of inhabitants with foreign migration background from the areas with the lowest proportion (< 10%) to the areas with the highest proportion (≥ 40%) of inhabitants with foreign backgrounds (Fig. 3). Between 2010 and 2019, there was a decrease of caries-free children in the third group (20%–29.9%) and an increase in the fourth group (30%–39.9%). The proportion of caries-free children in the areas with the lowest proportions of foreign migration background was 85% in 2010 and 84% in 2019 (*p* = 0.20), and 41% in 2010 and 46% (*p* = 0.15) in 2019 in the areas with the highest proportion; hence the difference in caries prevalence between the areas was 6pp less in 2019 than in 2010. The unadjusted OR for caries experience in areas with the highest proportion with foreign migration background in comparison to areas with the least was 6.3 in 2019 compared to 8.3 in 2010 (*p* = 0.07).

**Fig. 4:**
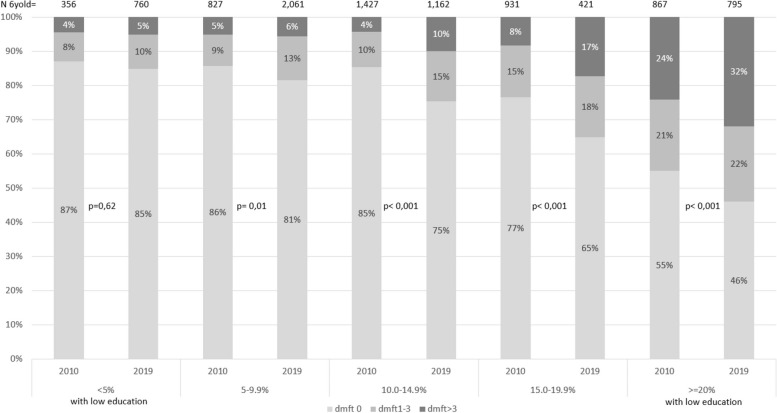
Caries prevalence by residential area based on the proportion of inhabitants with low educational level

The gradient was also evident when analysing groups in areas with increased proportions of inhabitants with low education (Fig. 4). Between 2010 and 2019, the proportion of caries-free children remained high; 87% in 2010 and 85% in 2019, in the areas with the least proportion of inhabitants with low education (< 5%). In the four other areas with a higher proportion of inhabitants with low education, the proportion of caries-free decreased and in the areas with the highest proportion (> 20%) the corresponding figures were 55% and 46% (*p* < 0.001). Hence the difference in caries prevalence between areas with the highest proportion of inhabitants with low education, compared to areas with the lowest proportion, was 7pp higher in 2019 compared to 2010. In 2010, the unadjusted OR for caries experience in areas with the highest proportions of inhabitants with low education was 5.5, in comparison to areas with the lowest proportions, and 6.6 in 2019 (*p* = 0.41).

## Discussion

The overall aim of this paper was to study 6-year-olds´ caries prevalence between 2010 and 2019 in relation to socioeconomic variables at family and residential area levels. The variables explaining the association between caries and socioeconomic variables were largely the same within each level and partly the same between the levels studied years. Despite changes in associations between studied years, the associations with caries prevalence and socioeconomic variables remained significant in both years. The overall results indicate that socioeconomic variables on both levels continued to be associated with caries in 6-year-olds and the associations were consistently stronger for severe caries experience than moderate, gradually increasing with higher levels of socioeconomic vulnerability.

When adjusting for confounders in the multivariable analysis, the models at both levels contained several explanatory variables in the association with caries, and children having multiple risk variables had an even higher likelihood of caries experience. At the family level, the explanatory socioeconomic variables were maternal age when the first child was born, maternal age when the child in the study group was born, parental employment, migration background of both parents and children, type of housing, and financial assistance (2010 only); and at area level migration background, parental educational level and number of persons per household. The ORs in the models were reduced compared to the univariable analysis. The results illustrate the complex relationship between the different socioeconomic variables and health [42].

On the family level, maternal age when the first child was born explained most of the association for both years and at the area level, mean parental age when having the first child was associated with caries in the univariable analysis. Maternal age at childbirth may not be a socioeconomic variable in and of itself but is associated with maternal socioeconomic situation and health outcome in offspring as well as being a risk indicator for caries, as both our and previous studies have shown [39, 43]. In our study, maternal age < 25 of the first child was associated with a higher likelihood of caries in the child and the associations were even stronger for mothers having the first child < 20 years of age. In addition, maternal age when having the child in the study group also showed an increased likelihood of severe caries if the mother's age was < 25 years, but also at > 34 years at childbirth. This finding is supported by an earlier study from Sweden demonstrating a U-shaped association between caries in children and maternal age, where mothers < 25 or > 35 years at childbirth had an increased risk of caries in their children [44]. The fact that the U-shape is not observed for the age at the first child may be because the number of children in the family is also a predictor for caries, as caries risk increases with higher birth order among siblings [45]; perhaps as this means that more children need to share the same limited parental resources [46].

At the area level, migration background explained most of the relationship in both years, followed by the educational variable. At the family level, children's and parents' migration backgrounds were associated with caries, as was maternal level of education. Our result is in line with an analysis performed by the National Board of Health and Welfare in Sweden of the oral health development in children of preschool age and the interaction between children's oral health and their social and demographic background between 2011 and 2019. [47] According to their analysis country of birth, education and income were the three factors most clearly associated with children's oral health. Their results showed that children whose parents were born abroad had almost three times the risk of suffering from caries compared to children of native-born parents [47].

At the area level, living in areas with an increased average number of persons per household was associated with a decreased likelihood of caries. At the family level, however, more than five people per household were associated with an increased likelihood of caries in the univariable analysis, which, as mentioned, previous studies also identified [44, 45]. The relative inconsistency of this, and other variables, between family and area levels, may be due to the heterogeneity within the areas. For example, in our study, areas with a higher average number of persons per household also had an increased proportion of inhabitants owning their own house, which was associated with a reduced likelihood of caries in the univariable analysis. In areas with many houses, in addition to families with children, many elderly people also live alone in their houses. In socially disadvantaged areas, there is a large proportion of apartments, where, in addition to families, there are also many students and single households, which lowers the average number per household in the area. This heterogeneity within areas may be the reason why the results at area and individual levels diverge to some extent.

Composite indices describing multiple socioeconomic conditions in an area have been argued to be more stable and robust [26, 48]. In our study, the CNI was used as a composite socioeconomic index at the area level. CNI is a well-used composite index for resource allocation that takes into account the composition of the inhabitants in an area based on several constituent variables (proportions of inhabitants within the area aged + 65 living alone, born abroad, unemployed, aged 16–64 years, single parent with children < 18, person who moved into the area) [20, 49]. In the univariable analysis, CNI stood out in terms of the likelihood of severe caries; however, when adding other variables, which were partly the ones that build up the CNI variable, CNI was not significant in the model.

The contextual social conditions in areas and the impact they have on the families living there—for example on children’s oral health—were highlighted in a recent study from the Netherlands [50]. After adjusting for individual and neighborhood characteristics neighborhood deprivation remained significantly associated with severe caries and low odds of visiting the dentist yearly in 6-year-olds. In the analysis of oral health development in children of preschool age made by the National Board of Health and Welfare in Sweden, however, no neighbourhood effects after adjusting for individual social factors were seen, suggesting family characteristics has a greater impact than the area on caries in 6-year-olds [47]. In our study, based on goodness of fit, the models on family level were better than the area level models at estimating caries prevalence. However, the National Board of Health and Welfare in Sweden also noted that in general, more children with an increased risk of caries live in vulnerable areas, as individual socioeconomic conditions are linked to an increased risk for caries; therefore, targeting efforts to these areas is a way to reach children at a greater risk of caries. Area-level socioeconomic status is useful for measuring geographical inequalities, for distributing and evaluating health preventive efforts and as a proxy for individual SES; for example, where individual data is lacking due to confidentiality [51]. However, when using area level this way it is important to acknowledge the described heterogenicity within the areas and that the association between socioeconomic prerequisites and dental caries is at a population level, which is not the caries situation on the family level [52].

On the family level, there was an increased proportion of caries-free 6-year-olds with low level of maternal education during the years studied. This improvement was not seen among the group of children with a foreign migration background. It is likely that the group of children with foreign migration background have had fewer opportunities to take part in the preventive measures offered by the region since they have lived in the region for a shorter time. This improvement in oral health among children at potentially higher risk of caries may indicate that the preventive measures offered by the region have reached these children. However, during the same period, there was an increased proportion of children with caries experience among children whose mothers’ highest attained education level was high school. This finding and the gradient in health found in our study, as well as in previous research, indicate the importance of oral health efforts being universal, to all, but also proportional, gradually increased with increased risk of disease [4].

A repeated cross-sectional study comparing time differences in caries-prevalence for 15-year-old Danish adolescents concluded persisting associations between socioeconomic position and dental caries from 1995 up until 2013 [53]. During that period, there was a steep decline in caries prevalence for all social groups in Denmark and a decrease in absolute inequality as the difference in caries prevalence between the groups decreased in both groups improving in terms of caries prevalence. However, relative inequality between different social groups had increased as the difference in likelihood of caries disease (OR) had increased. In our study, there was no general reduction in the prevalence of caries between the years studied—rather the opposite. However, for maternal level of education at the family level, both absolute and relative inequalities had decreased, as had the difference in the proportion of caries-free children and the likelihood of developing caries between children with the lowest and highest levels of maternal education. Despite these positive results, no change in absolute or relative inequality (or the opposite) was observed for migration background at either level or for the educational variable at the area level. Furthermore, the association between caries prevalence in 6-year-olds and the socioeconomic variables included in the models (except financial assistance) continued to be significantly associated with caries at both levels during the period studied. The result is in accordance with the Lancet series on oral health, declaring persistent oral health inequalities disproportionately affect poorer and marginalised groups in society [7, 54]. Furthermore, as caries in early childhood is a risk factor for poor oral health, continued caries disease and need for dental care as an adult, childhood caries can affect an individual throughout life and establish inequalities in health that persist over time [55, 56]. Oral health shares the same risk factors as other NCD closely linked to the social determinants of health—for example, diabetes and obesity—hence the development in caries prevalence in deciduous teeth may serve as a measurable indicator of these and for the development of health inequalities in society [8, 12, 57, 58]. In the Lancet series, Watt et al. argue that a fundamentally different approach than the current treatment-dominated approach is needed with upstream, midstream, and downstream policies and interventions targeting the underlying social determinants of health in joint action with the prevention of other NCDs [54].

The increase in caries prevalence among 6-year-olds between 2010 and 2019 observed at the family level in the present study was also observed nationwide [23]. During the same period, the proportion of children and parents with foreign migration backgrounds had increased in our study. The proportion with caries experience in the groups with foreign respective native migrant background remained unchanged but with a significantly larger proportion of 6-year-olds with caries experience in the group with a foreign migration background. As already described, caries prevalence is heavily skewed worldwide, ranging from 18.7% to 53.2% between different countries, and Sweden is among the countries with an average better oral health in the population [8]. The increase in caries prevalence among 6-year-olds during the studied period can therefore partly be explained by the population with foreign migration background being larger in 2019 than in 2010. In the national in-depth analysis of oral health development in children up to 6 years of age, it was also assessed that the increased proportion of children with a migrant background could partly explain the increase in caries prevalence in 6-year-olds between 2013 and 2019 but also difficulties in assessing caries risk in young children in combination with extended revision intervals for children who were assessed as low risk [47]. The results of this study show the importance of including social conditions when assessing caries risk and planning caries preventive interventions at the individual or area level, such as giving special attention to the oral health of children in migrant families or in areas where the proportion of migrant families is greater. In addition to the migration during this period income inequality and relative income poverty have increased in Sweden [59]. Increasing economic inequality has various and lasting effects on other forms of inequality in society such as an increase in poverty and health inequalities. This development may have contributed to the increase in caries prevalence among small children in Sweden.

Since Region Östergötland is the fourth largest region in Sweden, with both rural and urban areas, and since regional epidemiological caries data follow the national development, the results are considered representative of the Swedish population and can also be generalised to other countries with similar conditions as, for example, Scandinavian countries with similar publicly funded welfare systems. As the caries prevalence among small children in Sweden generally is low in an international comparison validation of this study on other populations would provide a more comprehensive picture of the development of oral health equality among children internationally [21].

Since this study was conducted, recent national data on caries prevalence has indicated that the negative trend in the decrease of caries-free children seems to have broken after 2019. In 2021, the proportion of caries-free 6-year-olds was 75% [23, 60]. However, national data and research have shown that health inequalities are at risk of worsening after the COVID-19 pandemic, as the pandemic affected individuals in vulnerable situations more than individuals in more favourable social conditions. The effects of the pandemic were both direct (disease and death), but also indirect through changes in living conditions, health habits, and through a reduced range of support and care opportunities in health care [61, 62]. The pandemic’s impact on health over time remains to be evaluated, but overall, this indicates that the risk of continued inequality remains [63].

### Strengths and weaknesses

A strength of this study was the coverage of the study population, where more than 94% of relevant individuals were included in the study. Reporting epidemiological caries data is mandatory in the remuneration system, contributing to the comprehensive coverage. However, there is a risk of bias, as 6-year-olds excluded from this study due to missing data may to a greater extent be children living in social vulnerability, as socially disadvantaged people are less likely to visit the dentist and are at greater risk of poorer oral health. This dropout may have affected the outcome of our study and underestimated the relationship between socioeconomic variables and caries [14, 50].

Despite extensive national registries at SCB there were missing values for several socioeconomic variables on the family level. Missing data could be unfortunate since there is an increased risk children living in social vulnerability could be overrepresented in this group. This was avoided by using multiple imputation to compensate for missing values at the family level. However, no multiple imputation was performed on area level as the only variables with missing values were paternal (*n* = 463) and maternal (*n* = 498) age when having the first child, and this was due to no first-born children being born in the areas studied years.

Socioeconomic variables can be strongly correlated with each other, which can cause multicollinearity that affects the results and makes it difficult to distinguish the individual effects of the included variables in the analysis. To avoid multicollinearity in the present study, VIF analyses were performed, and if two variables were highly correlated the variable with the least association with caries was removed.

The composition and limitations of the area may affect the results, and a low number of inhabitants may have caused uncertainty in the results. Neither health nor socioeconomic conditions follow administrative boundaries, and as described, this heterogeneity within areas can hide unequal conditions between, for example, socioeconomic groups within cities. NYKO4 was chosen as the definition of smaller areas based on the assumption that smaller areas expose more of the socioeconomic differences in society compared to larger areas. NYKO4 has the advantage of areas being defined and managed by the municipalities, which are well familiar with the social conditions and are rather small to expose more of the socioeconomic disparities.

## Conclusions

Identifying inequalities is important to make them visible and thereby provide the conditions to even them out. This study adds a long-term perspective on children’s oral health and the relationship between caries in children and socioeconomic conditions within families and areas. However, despite efforts directed to families and areas at greater risk of caries, and minor signs of improvement noted for children with low maternal level of education, the overall results indicate that socioeconomic variables on both family and area levels have continued to be associated with caries in 6-year-olds in Sweden. Associations were consistently stronger for severe caries experience than for moderate and were gradually increasing, with higher levels of socioeconomic vulnerability. Multiple socioeconomic risk variables meant an even greater likelihood of caries. For instance, the OR for severe caries for children with maternal age when the first child was born < 20 years, and with foreign maternal background was 6.0 compared to children with maternal age of 25–34 years and native maternal background. The results can be used to identify families with a higher caries risk in the individual risk assessment or to identify areas useful for targeted interventions. Future oral health strategies must be supportive of families in socially vulnerable contexts to even out health disparities and the persisting oral health inequalities indicate a different approach, targeting the social determinants of health, is needed. In addition, it is important to continue to monitor oral health based on socioeconomic conditions to ensure good and equal oral health. These persistent oral health disparities in caries risk follow children as they grow and affect both their oral and general health, cementing health inequalities.

## Supplementary Information

Supplementary Material 1.

Supplementary Material 2.

Supplementary Material 3.

Supplementary Material 4.

## Data Availability

The datasets used and/or analysed during the current study are available from the corresponding author on reasonable request.

## References

[1] Black D, Townsend P, N D. Inequalities in health: the Black report. Harmondsworth; 1982.

[2] Mackenbach JP, Stirbu I, Roskam AJ, Schaap MM, Menvielle G, Leinsalu M, et al. Socioeconomic inequalities in health in 22 European countries. N Engl J Med. 2008;358(23):2468–81. 18525043 10.1056/NEJMsa0707519

[3] WHO. Global Status Report on noncommunicable diseases 2014. https://www.who.int/publications/i/item/9789241564854 Access Date: 2024–04–19; 2014.

[4] Marmot M, Hlt CSD. Achieving health equity: from root causes to fair outcomes. Lancet. 2007;370(9593):1153–63. 17905168 10.1016/S0140-6736(07)61385-3

[5] WHO. A Conceptual Framework for Action on the Social Determinants of Health. https://www.who.int/publications/i/item/9789241500852 Access Date: 2024–04–19; 2010.

[6] Grad FP. The Preamble of the Constitution of the World Health Organization. Bull World Health Organ. 2002;80(12):981–4. 12571728 PMC2567708

[7] Peres MA, Macpherson LMD, Weyant RJ, Daly B, Venturelli R, Mathur MR, et al. Oral diseases: a global public health challenge. Lancet. 2019;394(10194):249–60. 31327369 10.1016/S0140-6736(19)31146-8

[8] WHO. Global oral health status report: towards universal health coverage for oral health by 2030. https://www.who.int/publications/i/item/9789240061484 Access Date: 2024–04–19; 2022.

[9] Kassebaum NJ, Smith AGC, Bernabe E, Fleming TD, Reynolds AE, Vos T, et al. Global, Regional, and National Prevalence, Incidence, and Disability-Adjusted Life Years for Oral Conditions for 195 Countries, 1990–2015: A Systematic Analysis for the Global Burden of Diseases, Injuries, and Risk Factors. J Dent Res. 2017;96(4):380–7. 28792274 10.1177/0022034517693566 PMC5912207

[10] Anil S, Anand PS. Early childhood caries: prevalence, risk factors, and prevention. Front Pediatr. 2017;5:157. 28770188 10.3389/fped.2017.00157 PMC5514393

[11] Andre Kramer AC, Skeie MS, Skaare AB, Espelid I, Ostberg AL. Caries increment in primary teeth from 3 to 6 years of age: a longitudinal study in Swedish children. Eur Arch Paediatr Dent. 2014;15(3):167–73. 24008371 10.1007/s40368-013-0079-7

[12] Ruiz B, Broadbent JM, Murray Thomson W, Ramrakha S, Boden J, Horwood J, et al. Is childhood oral health the 'canary in the coal mine' for poor adult general health? Findings from two New Zealand birth cohort studies. Community Dent Oral Epidemiol. 2022. 10.1111/cdoe.12772 36000812

[13] Fejerskov O, Nyvad B, Kidd E. Dental caries. The disease and its clinical management. 3rd ed. Oxford: Wiley-Blackwell; 2015.

[14] Splieth CH, Steffen H, Welk A, Schwahn C. Responder and nonresponder analysis for a caries prevention program. Caries Res. 2005;39(4):269–72. 15942185 10.1159/000084832

[15] Schou L, Wight C. Does dental health education affect inequalities in dental health? Community Dent Health. 1994;11(2):97–100. 8044719

[16] Davidson T, Blomma C, Bagesund M, Krevers B, Vall M, Warnberg Gerdin E, et al. Cost-effectiveness of caries preventive interventions - a systematic review. Acta Odontol Scand. 2020:1–12. 10.1080/00016357.2020.1862293 33370544

[17] Featherstone JDB, Crystal YO, Alston P, Chaffee BW, Domejean S, Rechmann P, et al. A Comparison of Four Caries Risk Assessment Methods. Front Oral Health. 2021;2: 656558. 35048004 10.3389/froh.2021.656558 PMC8757708

[18] Meijer M, Engholm G, Grittner U, Bloomfield K. A socioeconomic deprivation index for small areas in Denmark. Scand J Public Health. 2013;41(6):560–9. 23599378 10.1177/1403494813483937

[19] Hosseinpoor AR, Bergen N. Area-based units of analysis for strengthening health inequality monitoring. Bull World Health Organ. 2016;94(11):856–8. 27821889 10.2471/BLT.15.165266 PMC5096344

[20] Ostberg AL, Kjellstrom AN, Petzold M. The influence of social deprivation on dental caries in Swedish children and adolescents, as measured by an index for primary health care: The Care Need Index. Community Dent Oral Epidemiol. 2017;45(3):233–41. 28134453 10.1111/cdoe.12281

[21] Koch G, Helkimo AN, Ullbro C. Caries prevalence and distribution in individuals aged 3–20 years in Jonkoping, Sweden: trends over 40 years. Eur Arch Paediatr Dent. 2017;18(5):363–70. 28956292 10.1007/s40368-017-0305-9

[22] Swedish National Board of Health and Welfare in Sweden. [Caries in children and adolscents. Epidemiological data for 2010]. www.socialstyrelsen.se Access Date: 2024–04–09; 2011 2011–12–08.

[23] Swedish National Board of Health and Welfare in Sweden. [Caries in children and adolscents. Epidemiological data for 2019]. www.socialstyrelsen.se; 2021 2021–03–18.

[24] [The Dental Care Law (1985:125)], (1985).

[25] Guarnizo-Herreno CC, Watt RG, Pikhart H, Sheiham A, Tsakos G. Socioeconomic inequalities in oral health in different European welfare state regimes. J Epidemiol Community Health. 2013;67(9):728–35. 23814268 10.1136/jech-2013-202714

[26] Andre Kramer AC, Petzold M, Hakeberg M, Ostberg AL. Multiple socioeconomic factors and dental caries in swedish children and adolescents. Caries Res. 2018;52(1–2):42–50. 29237152 10.1159/000481411

[27] Schwendicke F, Dorfer CE, Schlattmann P, Foster Page L, Thomson WM, Paris S. Socioeconomic inequality and caries: a systematic review and meta-analysis. J Dent Res. 2015;94(1):10–8. 25394849 10.1177/0022034514557546

[28] Hou F, Myles J. Neighbourhood inequality, neighbourhood affluence and population health. Soc Sci Med. 2005;60(7):1557–69. 15652687 10.1016/j.socscimed.2004.08.033

[29] Swedish National Board of Health and Welfare in Sweden. [Social differences in dental health in children and young people. Background report to Children´s and young people´s health and care 2013]. https://www.socialstyrelsen.se/globalassets/sharepoint-dokument/artikelkatalog/ovrigt/2013-5-34.pdf Access Date: 24–04–09; 2013.

[30] Andre Kramer AC, Pivodic A, Hakeberg M, Ostberg AL. Multilevel Analysis of Dental Caries in Swedish Children and Adolescents in Relation to Socioeconomic Status. Caries Res. 2019;53(1):96–106. 30001533 10.1159/000489570

[31] Lundberg O. The next step towards more equity in health in Sweden: how can we close the gap in a generation? Scand J Public Health. 2018;46(22_suppl):19–27. 10.1177/1403494818765702 29862904

[32] WHO. Draft Global Oral Health Action Plan (2023–2030). https://www.who.int/publications/m/item/draft-global-oral-health-action-plan-%282023-2030%29 Access Date: 2024–04–19; 2022.

[33] Statistic Sweden (SCB). Statistical database 2023. https://www.statistikdatabasen.scb.se/pxweb/en/ssd/START__BE__BE0101/. Access Date 2023–01–24 [

[34] Mastrovito B, Blomma C, Johansson A, Borgstedt-Risberg M. [Follow-up on oral helath in children and young people in the region of Östergötland 1994–2019]. Linköping: Region Östergötland, Centrum för verksamhetsstöd och utveckling; 2020.

[35] Aronsson K, Mako E. [Dental health in children and adolescents in Östergötland municipalities 2010] Dental health report. Linköping: Folkhälsocentrum. Region Östergötland; 2011.

[36] Klein H, Palmer C, Knutson J. Studies on Dental Caries:1. Dental Status and Dental Needs of Elementary School Children. Public Health Reports 1938;53(1):751–65.

[37] WHO. Oral health surveys: basic methods - 5th edition. https://www.who.int/publications/i/item/9789241548649 Access Date: 2024–04–09; 2013 Access date: October 18, 2021.

[38] Statistic Sweden (SCB). [Statistics for small areas with keycode system (NYKO)] https://www.scb.se/contentassets/4d5516f7c4504a669fdf242136eacfee/meromnyko-.pdf Access Date: 2024–04–19: Statistics Sweden; https://www.scb.se/en/ [

[39] Julihn A, Soares FC, Hjern A, Dahllof G. Socioeconomic determinants, maternal health, and caries in young children. JDR Clin Trans Res. 2018;3(4):395–404. 30263967 10.1177/2380084418788066 PMC6139990

[40] Donders AR, van der Heijden GJ, Stijnen T, Moons KG. Review: a gentle introduction to imputation of missing values. J Clin Epidemiol. 2006;59(10):1087–91. 16980149 10.1016/j.jclinepi.2006.01.014

[41] Eekhout I, van de Wiel MA, Heymans MW. Methods for significance testing of categorical covariates in logistic regression models after multiple imputation: power and applicability analysis. BMC Med Res Methodol. 2017;17(1):129. 28830466 10.1186/s12874-017-0404-7 PMC5568368

[42] Eckersley R. Beyond inequality: Acknowledging the complexity of social determinants of health. Soc Sci Med. 2015;147:121–5. 26560411 10.1016/j.socscimed.2015.10.052

[43] Saloojee H, Coovadia H. Maternal age matters: for a lifetime, or longer. Lancet Glob Health. 2015;3(7):e342–3. 25999095 10.1016/S2214-109X(15)00034-0

[44] Soares FC, Dahllof G, Hjern A, Julihn A. U-shaped association between maternal age at delivery and dental caries in offspring. Acta Odontol Scand. 2020;78(8):565–71. 32363974 10.1080/00016357.2020.1756404

[45] Julihn A, Soares FC, Hammarfjord U, Hjern A, Dahllof G. Birth order is associated with caries development in young children: a register-based cohort study. BMC Public Health. 2020;20(1):218. 32050937 10.1186/s12889-020-8234-7 PMC7017501

[46] Downey DB. Number of siblings and intellectual development. The resource dilution explanation Am Psychol. 2001;56(6–7):497–504. 11413873 10.1037//0003-066x.56.6-7.497

[47] Swedish National Board of Health and Welfare in Sweden. [Oral health development among children in preschool age; The interaction between children's oral health and their social and demographic background]. https://www.socialstyrelsen.se/globalassets/sharepoint-dokument/artikelkatalog/ovrigt/2022-6-7991.pdf Access Date: 2024–04–19; 2022.

[48] Bajekal M, Jan S, Jarman B. The Swedish UPA score: an administrative tool for identification of underprivileged areas. Scand J Soc Med. 1996;24(3):177–84. 8878371 10.1177/140349489602400309

[49] Sundquist K, Malmstrom M, Johansson SE, Sundquist J. Care Need Index, a useful tool for the distribution of primary health care resources. J Epidemiol Community Health. 2003;57(5):347–52. 12700218 10.1136/jech.57.5.347 PMC1732439

[50] van Meijeren-van Lunteren AW, Oude Groeniger J, Wolvius EB, Kragt L. Neighbourhood characteristics and children’s oral health: a multilevel population-based cohort study. Eur J Public Health. 2021;31(4):742–8. 33624096 10.1093/eurpub/ckab013 PMC8514066

[51] Galobardes B, Lynch J, Smith GD. Measuring socioeconomic position in health research. Br Med Bull. 2007;81–82:21–37. 17284541 10.1093/bmb/ldm001

[52] Piantadosi S, Byar DP, Green SB. The ecological fallacy. Am J Epidemiol. 1988;127(5):893–904. 3282433 10.1093/oxfordjournals.aje.a114892

[53] Sengupta K, Christensen LB, Mortensen LH, Skovgaard LT, Andersen I. Trends in socioeconomic inequalities in oral health among 15-year-old Danish adolescents during 1995–2013: A nationwide, register-based, repeated cross-sectional study. Community Dent Oral Epidemiol. 2017;45(5):458–68. 28653759 10.1111/cdoe.12310

[54] Watt RG, Daly B, Allison P, Macpherson LMD, Venturelli R, Listl S, et al. Ending the neglect of global oral health: time for radical action. Lancet. 2019;394(10194):261–72. 31327370 10.1016/S0140-6736(19)31133-X

[55] Isaksson H, Alm A, Koch G, Birkhed D, Wendt LK. Caries prevalence in Swedish 20-year-olds in relation to their previous caries experience. Caries Res. 2013;47(3):234–42. 23328627 10.1159/000346131

[56] Peres MA, Peres KG, de Barros AJ, Victora CG. The relation between family socioeconomic trajectories from childhood to adolescence and dental caries and associated oral behaviours. J Epidemiol Community Health. 2007;61(2):141–5. 17234873 10.1136/jech.2005.044818 PMC2465630

[57] Watt RG, Mathur MR, Aida J, Bonecker M, Venturelli R, Gansky SA. Oral Health Disparities in Children: A Canary in the Coalmine? Pediatr Clin North Am. 2018;65(5):965–79. 30213357 10.1016/j.pcl.2018.05.006

[58] G B D Risk Factors Collaborators. Global, regional, and national comparative risk assessment of 79 behavioural, environmental and occupational, and metabolic risks or clusters of risks, 1990–2015: a systematic analysis for the Global Burden of Disease Study 2015. Lancet. 2016;388(10053):1659–724. 10.1016/S0140-6736(16)31679-8 PMC5388856 27733284

[59] Sweden's economic inequality gap is widening and worrying. The Lancet Regional Health - Europe. 2023;26(100610). 10.1016/j.lanepe.2023.100610 PMC9989655 36895448

[60] Swedish National Board of Health and Welfare. [Caries in children and adolscents. Epidemiological data for 2021] 2022–5–7906. https://www.socialstyrelsen.se/en/; 2022.

[61] The Public Health Agency of Sweden. [Public Health in Sweden. Annual Report 2023]. https://www.folkhalsomyndigheten.se/contentassets/a448b27d603c44f590fc1aff741b0d5d/folkhalsan-sverige-arsrapport-2023.pdf Access Date: 2024–04–19; 2023.

[62] Stennett M, Tsakos G. The impact of the COVID-19 pandemic on oral health inequalities and access to oral healthcare in England. Br Dent J. 2022;232(2):109–14. 35091614 10.1038/s41415-021-3718-0 PMC8796193

[63] Swedish National Board of Health and Welfare in Sweden. [Effects of COVID-19 on oral health and dental visits among children and Adults – Part 4 ]. https://www.socialstyrelsen.se/globalassets/sharepoint-dokument/artikelkatalog/ovrigt/2022-5-7887.pdf Access Date: 2024–04–19; 2022. Contract No.: 2022–5–7887.

