# Supplementary material for: Persistent oral health inequality in children—repeated cross-sectional studies in 2010 and 2019
Source: BMC Public Health. 2024 Dec 18;24:3528. doi: 10.1186/s12889-024-20905-y (PMC11658173; doi:10.1186/s12889-024-20905-y)
Supplement: Supplementary file 1 — Supplementary Material 1. [file 12889_2024_20905_MOESM1_ESM.docx]

Appendix 1. Independent variables and associated categories:

| Individual/ family level | | Residential area level | |
| --- | --- | --- | --- |
| (refers to 20101231 resp 20191231) | | **(refers to y2010 resp y2019 if nothing else is specified bellow)** | |
| Variable | **Categories** | **Variable** | **Categories** |
| Cohort childs year of birth (N of children in each cohort) | 2010 | Population age | Proportion of inhabitants age <7 |
|  | 2019 |  | 7-18 |
|  |  |  | 19-64 |
|  |  |  | >65 |
| Childs gender | Male | Gender | Proportion of male |
|  | Female |  |  |
| Childs´ ethnicity | Born in Sweden | Ethnicity | Proportion born in Sweden |
|  | Born in Europe except Sweden |  | Proportion born outside Sweden |
|  | Born outside Europe |  |  |
| Childs´ and parental (maternal and paternal) migration background (Refers to birth country and parents country of origin) | “Born in Sweden with one or two native Swedish parents” = native | Migration background | “Born in Sweden with one or two native Swedish parents”= proportion with native background |
|  | “Born abroad or born in Sweden with two foreign  born parents”= foreign |  | “Born abroad or born in Sweden with two foreign  born parents”= foreign background |
| Parental (maternal and paternal) age when the child in the study group was born | Adolescent =<20y |  |  |
|  | Young 20-24y |  |  |
|  | Middle-aged 25-34y |  |  |
|  | Older >35y |  |  |
| Parental (maternal and paternal) age when their first child was born | Adolescent =<20y | Parental age when first child was born | Average age when first child was born |
|  | Young 20-24y |  |  |
|  | Middle-aged 25-34y |  |  |
|  | Older >35y |  |  |
| Parental (maternal and paternal) employment status | “worked all year”= employed, “worked occasionally or not at all”= unemployed | Employment | Proportion of employment |
| Parental (maternal and paternal) educational level (Sun2000Niva_Old) | ≤9 years=elementary school | Educational level | Proportion of inhabitants (16-74y) at each education**al** level (≤9 years=elementary school, |
|  | Highschool education |  | Highschool education |
|  | Higher education |  | Higher education |
|  |  |  | Else |
| Family type | Singel parent with at least one child <18 living in the household | Family type (2011/2019) | Proportion of single mothers/ fathers |
|  | Not singel parent with at least one child |  |  |
|  | Else= children 15 years and younger registered as single<18 living in the household |  |  |
| Number of children/ household | Only child=1, | Number of children/ household (2011/2019) | Average number of children/ household |
|  | 2-3 children, |  |  |
|  | 4 children or more |  |  |
| Number of persons/ household | 1-5 persons | Number of persons/ household (2011/2019) | Average number of persons/ household |
|  | >5 persons |  |  |
| Family´s disposable income* | Bellow the lowest quintile "low income" (y 2010 317350 SEK, y 2019 352900 SEK) | Disposable income * (2011/2019) | Median disposable income per household |
|  | Above the highest quarter "high income" (y 2010 622050 SEK, y 2019 717000 SEK) |  | Median disposable income per consumption unit/ household |
|  | In between |  |  |
|  |  | Proportion of high-income households | Proportion of households whose disposable income belongs to the top quarter for disposable income (>352 255 y 2019, >280 526 y 2011) |
| Financial assistance | Yes | Financial assistance (2011/2019) | Proportion of households with financial assistance |
|  | No |  |  |
| Housing allowance | Yes | Housing allowance | Proportion of households with housing allowance |
|  | No |  |  |
| Form of housing | Renting | Form of housing (2012/2019) | Proportion living at each form of housing renting |
|  | Owning apartment in apartment building |  | Owning apartment in apartment building |
|  | Owning house |  | Owning house |
|  | Else (eg nursing homes or special housing) |  | Else |
| Family living in in urban or rural area | Rural | Area urban/rural (2011/2019) | Proportion of households in urban |
|  | Urban |  | Rural area |
|  |  | Residential area Care Need Index (CNI) | 0-1 |
|  |  |  | >1 |

*Disposable income for 2010 was converted into the 2019s´ index (conversion variable 2010-2019 1,10).
