# Supplementary material for: Persistent oral health inequality in children—repeated cross-sectional studies in 2010 and 2019
Source: BMC Public Health. 2024 Dec 18;24:3528. doi: 10.1186/s12889-024-20905-y (PMC11658173; doi:10.1186/s12889-024-20905-y)
Supplement: Supplementary file 2 — Supplementary Material 2. [file 12889_2024_20905_MOESM2_ESM.zip › Appendix 2a.docx]

Appendix 2a. Descriptive data on caries prevalence and socioeconomic variables at family level, for year 2010 and 2019 and significant differences in proportions between the years

|  |  | 2010 | 2019 |  |
| --- | --- | --- | --- | --- |
| Variable | Category | n (%) | n (%) | *p-*value |
| 6y |  | 4,408 | 5,199 |  |
| Caries (dmft) | 0 | 3,426 (77.7) | 3,838 (73.8) |  |
|  | 1-3 | 574 (13.0) | 764 (14.7) |  |
|  | >3 | 408 (9.3) | 597 (11.5) | <0.001 |
| Childs gender | Male | 2,260 (51.3) | 2,691 (51.8) |  |
|  | Female | 2,148 (48.7) | 2,508 (48.2) | 0.632 |
| Childs´ ethnicity | Sweden | 4,156 (94.3) | 4,813 (92.6) |  |
|  | European | 54(1.2) | 84 (1.6) |  |
|  | Outside of Europe | 198 (4.5) | 301 (5.8) | <0.01 |
|  | Missing | - | 1 |  |
| Childs´ migration background | Native | 3,678 (83.4) | 3,998 (76.9) |  |
|  | Foreign | 730 (16.6) | 1201 (23.1) | <0.001 |
| Maternal migration background | Native | 3,438 (78.2) | 3,617 (69.7) |  |
|  | Foreign | 959 (21.8) | 1,573 (30.3) | <0.001 |
|  | Missing | 11 | 9 |  |
| Paternal migration background | Native | 3,394 (78.3) | 3,541 (69.7) |  |
|  | Foreign | 942 (21.7) | 1,537 (30.3) | <0.001 |
|  | Missing | 72 | 121 |  |
| Maternal age when child in the study group was born | Mean | 30.1 | 30.3 |  |
|  | Range | 15-48 | 15-48 |  |
|  | <20y | 76 (1.7) | 64 (1.2) |  |
|  | 20-24y | 530 (12.0) | 686 (13.2) |  |
|  | 25-34y | 2,970 (67.4) | 3,291 (63.4) |  |
|  | >35y | 829 (18.8) | 1,152 (22.2) | <0.001 |
|  | Missing | 3 | 6 |  |
| Paternal age when child in the study group was born | Mean age | 32.9 | 33.3 |  |
|  | Range age | 14-64 | 16-73 |  |
|  | <20y | 29 (0.7) | 19 (0.4) |  |
|  | 20-24y | 208 (4.8) | 326 (6.4) |  |
|  | 25-34y | 2,604 (59.6) | 2,770 (54.4) |  |
|  | >35y | 1,528 (35.0) | 1,977 (38.8) | <0.001 |
| Maternal age when first child was born | Missing | 39 | 107 |  |
|  | Mean | 27.0 | 27.1 |  |
|  | Range | 14-44 | 13-47 |  |
|  | <20y | 295 (6.7) | 300 (5.8) |  |
|  | 20-24y | 1,100 (25.0) | 1,356 (26.1) |  |
|  | 25-34y | 2,726 (62.0) | 3,145 (60.6) |  |
|  | >35y | 276 (6.3) | 389 (7.5) | <0.01 |
|  | Missing | 11 | 9 |  |
| Paternal age when their first child was born | Mean | 29.5 | 29.7 |  |
|  | Range | 14-62 | 15-59 |  |
|  | <20y | 70 (1.6) | 85 (1.7) |  |
|  | 20-24y | 630 (14.5) | 749 (14.7) |  |
|  | 25-34y | 2,953 (68.1) | 3,359 (66.1) | 0.144 |
|  | Missing | 72 | 121 |  |
| Maternal employment status | Employed | 3,475 (79.0) | 4,221 (81.4) |  |
|  | Unemployed | 922 (21.0) | 965 (18.6) | <0.01 |
|  | Missing | 11 | 13 |  |
| Maternal educational level | Elementary school | 446 (10.3) | 601 (13.7) |  |
|  | Highschool | 1,889 (43.4) | 1,676 (38.2) |  |
|  | Higher education | 2,015 (46.3) | 2,110 (48.1) | <0.001 |
|  | Missing | 58 | 812 |  |
| Paternal educational level | Elementary school | 460 (10.7) | 637 (14.2) |  |
|  | Highschool | 2,186 (50.7) | 2,116 (47.3) |  |
|  | Higher education | 1,669 (38.7) | 1,722 (38.5) | <0.001 |
|  | Missing | 93 | 724 |  |
| Family type | Single parent | 738 (16.7) | 968 (18.6) |  |
|  | Not single | 3,668 (83.3) | 4,226 (81.4) | 0.016 |
|  | Else | 2 | 5 |  |
| Number of children in the household | 1 | 465 (10.6) | 609 (11.7) |  |
|  | 2-3 | 3,499 (79.4) | 3,938 (75.8) |  |
|  | ≥4 | 442 (10.0) | 647 (12.5) | <0.001 |
|  | Missing | 2 | 5 |  |
| Number of persons/ household | 1-5 | 3,997 (91.1) | 3,999 (98.7) |  |
|  | >5 | 392 (8.9) | 52 (1.3) | <0.001 |
|  | Missing | 19 | 1148 |  |
| Familys´ disposable  income | Mean | 517,645 | 583,070 |  |
|  | Median | 506,770 | 572,050 | <0.001 |
|  | Missing | 19 | 21 |  |
| Financial  assistance | Yes | 385 (8.8) | 176 (5.9) |  |
|  | No | 4,004 (91.2) | 5,002 (96.6) | <0.001 |
|  | Missing | 19 | 21 |  |
| Housing allowance | No | 3,656 (82.9) | 4,246 (82.0) |  |
|  | Yes | 733 (16.7) | 932 (18.0) | 0.095 |
|  | Missing | 19 | 21 |  |
| Form of housing | Renting | 1,186(26.6) | 965(23.8) |  |
|  | Owning appartment | 159(3.6) | 186(4.6) |  |
|  | Owning house | 3,022(68.9) | 2,840(70.2) | <0.01 |
|  | Else | 40 | 56 |  |
|  | Missing | 19 | 1152 |  |
| Family living in in urban or rural | Urban | 3,707 (84.1) | 4,481 (86.2) |  |
|  | Rural | 701 (15.9) | 718 (13.8) | 0.004 |

^1^ native= Born in Sweden with one or two native Swedish parents

^2^ foreign=Born abroad or born in Sweden with two foreign born parents
