# Supplementary material for: Persistent oral health inequality in children—repeated cross-sectional studies in 2010 and 2019
Source: BMC Public Health. 2024 Dec 18;24:3528. doi: 10.1186/s12889-024-20905-y (PMC11658173; doi:10.1186/s12889-024-20905-y)
Supplement: Supplementary file 2 — Supplementary Material 2. [file 12889_2024_20905_MOESM2_ESM.zip › Appendix 2b.docx]

Appendix 2b. Descriptive data on caries prevalence and socioeconomic variables at residential area level, for year 2010 and 2019 and significant differences in proportions between the years.

|  |  | 2010 | 2019 |  |
| --- | --- | --- | --- | --- |
|  | Category | n (%) | n (%) | *P-*value |
| NYKO |  | 622 | 651 |  |
| N 6y-old/NYKO | Mean | 7.1 | 8.0 | 0.080 |
|  | Range | 1- 64 | 1- 71 |  |
|  | Std.Dev | 8.4 | 9.9 |  |
| Population | Mean | 658 | 685 | 0.506 |
|  | Range | 11-4,298 | 4-4,932 |  |
|  | Std.Dev | 703 | 746 |  |
| Proportion age 0-6 | Mean | 8.5 | 8.4 | 0.594 |
|  | Range | 1.7-38.7 | 1.4-50.0 |  |
|  | Std.Dev | 3.6 | 3.1 |  |
| Proportion age  7-18 | Mean | 14.6 | 14.0 | 0.023 |
|  | Range | 0.0-30.0 | 0.0-36.3 |  |
|  | Std.Dev | 4.8 | 5.3 |  |
| Proportion age 19-64 | Mean | 58.4 | 56.0 | <0.001 |
|  | Range | 26.7-89.2 | 27.9-85.8 |  |
|  | Std.Dev | 7.2 | 7.6 |  |
| Proportion age >65 | Mean | 18.5 | 21.7 | <0.001 |
|  | Range | 0.0-66.7 | 0.0-68.3 |  |
|  | Std.Dev | 8.6 | 9.1 |  |
| Caries  (Mean dmft/ NYKO) | Mean | 0.74 | 0.85 | 0.148 |
|  | Range | 0.0- 10.0 | 0.0- 10.5 |  |
|  | Std.Dev | 1.31 | 1.35 |  |
| Proportion of male | Mean | 50.7 | 51. 1 | 0.039 |
|  | Range | 36.0-72.7 | 37.5-72.7 |  |
|  | Std.Dev | 3.4 | 3.1 |  |
| Proportion born in Sweden | Mean | 91.3 | 88.3 | <0.001 |
|  | Range | 32.4-100.0 | 20.0-100.0 |  |
|  | Std.Dev | 8.6 | 11.3 |  |
| Proportion born outside Sweden | Mean | 8.7 | 11.7 | <0.001 |
|  | Range | 0-67.7 | 0-80.0 |  |
|  | Std.Dev | 8.6 | 11.3 |  |
| Proportion with native migration background | Mean | 88.8 | 84.7 | <0.001 |
|  | Range | 20.6-100.0 | 0.0-100.0 |  |
|  | Std.Dev | 11.2 | 14.6 |  |
| Proportion with foreign migration  background | Mean | 11.2 | 15.3 | <0.001 |
|  | Range | 0.0- 79.4 | 0.0-100.0 |  |
|  | Std.Dev | 11.2 | 14.9 |  |
| Average age of mothers at the birth of their first child | Mean | 27.6 | 28.4 | <0.01 |
|  | Range | 17-41 | 19-41 |  |
|  | Std.Dev | 3.7 | 3.4 |  |
|  | Missing | 179 | 207 |  |
| Average age of fathers at the birth of their first child | Mean | 30.4 | 30.4 | 0.965 |
|  | Range | 20-51 | 20-51 |  |
|  | Std.Dev | 3.8 | 3.9 |  |
|  | Missing | 182 | 208 |  |
| Proportion with employment | Mean | 50.2 | 51.0 | 0.045 |
|  | Range | 21.3-73.4 | 0.0-73.5 |  |
|  | Std.Dev | 7.1 | 7.6 |  |
| Proportion with highest education elementary school | Mean | 14.5 | 10.8 | <0.001 |
|  | Range | 0.0-39.0 | 0.0-42.1 |  |
|  | Std.Dev | 6.6 | 6.7 |  |
| Proportion with highest education high school | Mean | 50.3 | 48.4 | <0.01 |
|  | Range | 8.3-73.3 | 0.0-81.8 |  |
|  | Std.Dev | 10.3 | 11.5 |  |
| Proportion with higher education | Mean | 33.7 | 38.9 | <0.001 |
|  | Range | 5.4-91.7 | 0.0-100.0 |  |
|  | Std.Dev | 14.1 | 14.5 |  |
| Else | Mean | 1.4 | 2.0 |  |
|  |  |  |  |  |
| Proportion of single mothers | Mean | 4.0 | 3.6 | 0.045 |
|  | Range | 0.0-40.0 | 0.0-25.0 |  |
|  | Std.Dev | 3.6 | 3.0 |  |
| Proportion of single fathers | Mean | 1.5 | 1.7 | 0.066 |
|  | Range | 0.0-20.0 | 0.0-16.7 |  |
|  | Std.Dev | 1.7 | 1.6 |  |
| Average number of children/ household | Mean | 0.69 | 0.66 | 0.026 |
|  | Range | 0.04-1.75 | 0.04-2.00 |  |
|  | Std.Dev | 0.28 | 0.29 |  |
|  | Missing | 0 | 1 |  |
| Average number of persons/ household | Mean | 2.4 | 2.3 | 0.066 |
|  | Range | 1.3-3.8 | 1.3-4.0 |  |
|  | Std.Dev | 0.43 | 0.43 |  |
|  | Missing | - | 1 |  |
| Median disposable income/ household | Mean | 406,015 | 450,842 | <0.001 |
|  | Range | 112,565-687,921 | 134,768-1123,666 |  |
|  | Std.Dev | 117,411 | 135,465 |  |
|  | Missing | 0 | 1 |  |
| Median disposable income per consumption unit/ household | Mean | 233,007 | 450,842 | <0.001 |
|  | Range | 104,424-393,913 | 134,768-1,123,667 |  |
|  | Std.Dev | 38,829 | 135,465 |  |
|  | Missing | - | 1 |  |
| Proportion of high-income households | Mean | 10.8 | 11.1 | 0.1 |
|  | Range | 0.0-30.8 | 0.0-27.3 |  |
|  | Std.Dev | 4.3 | 4.4 |  |
| Proportion of households with financial assistance | Mean | 3.6 | 2.4 | <0.001 |
|  | Range | 0.0-43.5 | 0.0-30.6 |  |
|  | Std.Dev | 5.7 | 4.6 |  |
| Proportion of households with housing allowance | Mean | 4.2 | 3.8 | 0.310 |
|  | Range | 0.0-43.5 | 0.00-37.6 |  |
|  | Std.Dev | 6.3 | 6.0 |  |
| Proportions housing renting | Mean | 21.7 | 21.9 | 0.899 |
|  | Range | 0-100 | 0-100 |  |
|  | Std.Dev | 30.1 | 30.1 |  |
| Proportion owning appartment in appartment building | Mean | 6.5 | 7.6 | 0.285 |
|  | Range | 0-100 | 0-100 |  |
|  | Std.Dev | 17.0 | 17.9 |  |
| Proportion owning house | Mean | 66.0 | 66.2 | 0.91 |
|  | Range | 0-100 | 0-100 |  |
|  | Std.Dev | 36.7 | 38.1 |  |
|  | Missing | - | 1 |  |
| Else | Mean | 5.8 | 4.3 |  |
| Proportion of households in urban area | Mean | 65.7 | 66.3 | 0.824 |
|  | Range | 0-100 | 0-100 |  |
|  | Std.Dev | 46.1 | 45.8 |  |
|  | Missing | - | 1 |  |
| Residential area CNI | Mean | 1.14 | 1.19 | 0.148 |
|  | Range | 0.3-4.0 | 0.2-6.3 |  |
|  | Std.Dev | 0.5 | 0.7 |  |
