# Supplementary material for: Persistent oral health inequality in children—repeated cross-sectional studies in 2010 and 2019
Source: BMC Public Health. 2024 Dec 18;24:3528. doi: 10.1186/s12889-024-20905-y (PMC11658173; doi:10.1186/s12889-024-20905-y)
Supplement: Supplementary file 3 — Supplementary Material 3. [file 12889_2024_20905_MOESM3_ESM.docx]

Appendix 3. Univariable analysis of the association between caries and socioeconomic variables on family level year 2010 respectively 2019 for moderate and severe caries and the interaction over time.

|  |  |  | Comparison 1-3 against 0 | | | | | Comparison >3 against 0 | | | | |
| --- | --- | --- | --- | --- | --- | --- | --- | --- | --- | --- | --- | --- |
| Variable  (ref) | **Year** |  | ***p-*value** | **OR** | **Lower** | **Upper** | ***p*-interaction** | ***p*-value** | **OR** | **Lower** | **Upper** | ***p*-interaction** |
| Childs gender  (female) | 2010 | Male | 0.20 | 1.12 | 0.94 | 1.34 |  | 0.05 | 1.23 | 1 | 1.52 |  |
|  | 2019 | Male | 0.08 | 1.15 | 0.98 | 1.34 | 0.84 | 0.26 | 1.10 | 0.93 | 1.31 | 0.42 |
| Childs´ ethnicity (native) | 2010 | Europe | 0.16 | 1.76 | 0.80 | 3.88 |  | <0.001 | 6.18 | 3.36 | 11.37 |  |
|  |  | Outside Europe | <0.001 | 3.87 | 2.65 | 5.64 |  | <0.001 | 10.54 | 7.52 | 14.79 |  |
|  | 2019 | Europe | 0.76 | 1.12 | 0.54 | 2.31 | 0.71 | <0.001 | 6.45 | 4.04 | 10.30 | 0.68 |
|  |  | Outside Europe | <0.001 | 3.72 | 2.69 | 5.14 |  | <0.001 | 12.77 | 9.66 | 16.89 |  |
| Childs´ migration background (native) | 2010 | Foreign | <0.001 | 3.96 | 3.20 | 4.92 |  | <0.001 | 16.01 | 12.71 | 20.17 |  |
|  | 2019 | Foreign | <0.001 | 3.39 | 2.84 | 4.03 | 0.27 | <0.001 | 14.12 | 11.60 | 17.18 | 0.42 |
| Maternal migration background (native) | 2010 | Foreign | <0.001 | 3.08 | 2.53 | 3.76 |  | <0.001 | 11.87 | 9.45 | 14.92 |  |
|  | 2019 | Foreign | <0.001 | 3.05 | 2.59 | 3.59 | 0.96 | <0.001 | 11.73 | 9.59 | 14.36 | 0.94 |
| Paternal migration background (native) | 2010 | Foreign | <0.001 | 3.16 | 2.60 | 3.85 |  | <0.001 | 10.47 | 8.31 | 13.19 |  |
|  | 2019 | Foreign | <0.001 | 2.83 | 2.40 | 3.33 | 0.37 | <0.001 | 11.07 | 9.04 | 13.56 | 0.76 |
| Maternal age when child in the study group was born (25-34y ) | 2010 | <20y | <0.01 | 2.62 | 1.40 | 4.92 |  | <0.001 | 9.17 | 5.44 | 15.48 |  |
|  |  | 20-24y | <0.001 | 1.71 | 1.32 | 2.22 |  | <0.001 | 3.45 | 2.64 | 4.50 |  |
|  |  | >35y | 0.21 | 1.16 | 0.92 | 1.46 |  | <0.01 | 1.48 | 1.14 | 1.95 |  |
|  | 2019 | <20y | <0.01 | 2.49 | 1.28 | 4.87 | 0.66 | <0.001 | 6.32 | 3.62 | 11.03 | <0.01 |
|  |  | 20-24y | <0.01 | 1.44 | 1.15 | 1.81 |  | <0.001 | 2.12 | 1.69 | 2.66 |  |
|  |  | >35y | 0.96 | 1.00 | 0.82 | 1.21 |  | 0.058 | 0.80 | 0.63 | 1.01 |  |
| Paternal age when child in the study group was born (25-34y) | 2010 | <20y | 0.99 | 0.99 | 0.26 | 3.80 |  | <0.001 | 8.34 | 4.10 | 16.70 |  |
|  |  | 20-24y | 0.06 | 1.47 | 0.99 | 2.19 |  | <0.001 | 2.44 | 1.63 | 3.67 |  |
|  |  | >35y | 0.30 | 1.11 | 0.92 | 1.34 |  | 0.011 | 1.34 | 1.07 | 1.68 |  |
|  | 2019 | <20y | 0.05 | 2.43 | 1.00 | 5.90 | 0.53 | 0.026 | 2.93 | 1.14 | 7.49 | 0.13 |
|  |  | 20-24y | <0.01 | 1.51 | 1.11 | 2.06 |  | <0.01 | 1.71 | 1.23 | 2.38 |  |
|  |  | >35y | 0.08 | 1.16 | 0.98 | 1.38 |  | 0.058 | 1.20 | 0.99 | 1.45 |  |
| Maternal age when first child was born  (25-34y) | 2010 | <20y | <0.001 | 3.15 | 2.26 | 4.39 |  | <0.001 | 12.53 | 9.15 | 17.17 |  |
|  |  | 20-24y | <0.001 | 2.17 | 1.78 | 2.65 |  | <0.001 | 4.04 | 3.15 | 5.18 |  |
|  |  | >35y | 0.50 | 0.86 | 0.56 | 1.33 |  | 0.12 | 1.49 | 0.90 | 2.46 |  |
|  | 2019 | <20y | <0.001 | 2.96 | 2.17 | 4.05 | 0.83 | <0.001 | 7.74 | 5.76 | 10.41 | 0.03 |
|  |  | 20-24y | <0.001 | 1.92 | 1.61 | 2.28 |  | <0.001 | 3.65 | 3.00 | 4.44 |  |
|  |  | >35y | 0.32 | 0.85 | 0.61 | 1.18 |  | 0.09 | 0.65 | 0.39 | 1.07 |  |
| Paternal age when their first child was born (25-34y) | 2010 | <20y | 0.05 | 1.91 | 0.99 | 3.66 |  | <0.001 | 7.40 | 4.41 | 12.41 |  |
|  |  | 20-24y | <0.001 | 1.75 | 1.38 | 2.21 |  | <0.001 | 3.20 | 2.48 | 4.12 |  |
|  |  | >35y | 0.29 | 0.87 | 0.67 | 1.13 |  | 0.204 | 1.22 | 0.90 | 1.66 |  |
|  | 2019 | <20y | 0.01 | 1.97 | 1.16 | 3.34 | 0.21 | <0.001 | 2.72 | 1.65 | 4.49 | 0.01 |
|  |  | 20-24y | <0.001 | 1.68 | 1.36 | 2.08 |  | <0.001 | 2.26 | 1.82 | 2.82 |  |
|  |  | >35y | 0.07 | 1.21 | 0.99 | 1.49 |  | 0.747 | 1.04 | 0.81 | 1.34 |  |
| Maternal employment status (Yes) | 2010 | No | <0.001 | 2.83 | 2.32 | 3.45 |  | <0.001 | 8.65 | 6.94 | 10.77 |  |
|  | 2019 | No | <0.001 | 2.80 | 2.32 | 3.36 | 0.94 | <0.001 | 6.82 | 5.65 | 8.23 | 0,11 |
| Paternal employment status (Yes) | 2010 | No | <0.001 | 2.46 | 1.91 | 3.17 |  | <0.001 | 8.41 | 6.61 | 10.69 |  |
|  | 2019 | No | <0.001 | 2.49 | 1.95 | 3.18 | 0.89 | <0.001 | 6.46 | 5.22 | 8.00 | 0.12 |
| Maternal educational level  (Higher education) | 2010 | Elementary school | <0.001 | 3.94 | 2.98 | 5.22 |  | <0.001 | 14.99 | 11.15 | 20.16 |  |
|  |  | Highschool | <0.001 | 1.54 | 1.27 | 1.87 |  | <0.001 | 1.89 | 1.44 | 2.49 |  |
|  | 2019 | Elementary school | <0.001 | 2.94 | 2.30 | 3.76 | 0.11 | <0.001 | 8.01 | 5.34 | 12.03 | <0.001 |
|  |  | Highschool | <0.001 | 1.65 | 1.35 | 2.02 |  | <0.001 | 2.08 | 1.60 | 2.72 |  |
| Paternal educational level  (Higher education) | 2010 | Elementary school | <0.001 | 2.79 | 2.09 | 3.74 |  | <0.001 | 5.88 | 4.29 | 8.06 |  |
|  |  | Highschool | <0.001 | 1.45 | 1.19 | 1.78 |  | 0.051 | 1.31 | 1.00 | 1.71 |  |
|  | 2019 | Elementary school | <0.001 | 2.78 | 2.11 | 3.66 | 0.86 | <0.001 | 5.01 | 3.94 | 6.36 | 0.59 |
|  |  | Highschool | <0.01 | 1.39 | 1.13 | 1.71 |  | 0.09 | 1.22 | 0.97 | 1.532 |  |
| Family type  (not singel) | 2010 | Single | <0.01 | 1.39 | 1.11 | 1.75 |  | <0.001 | 2.13 | 1.68 | 2.69 |  |
|  | 2019 | Single | <0.001 | 1.50 | 1.25 | 1.81 | 0.61 | <0.01 | 1.33 | 1.08 | 1.65 | <0.01 |
| Number of children in the household (2-3) | 2010 | 1 | 0.12 | 1.25 | 0.95 | 1.66 |  | 0.21 | 1.25 | 0.88 | 1.78 |  |
|  |  | >3 | <0.001 | 2.34 | 1.79 | 3.06 | 0.58 | <0.001 | 5.22 | 4.04 | 6.75 | 0.15 |
|  | 2019 | 1 | 0.07 | 1.25 | 0.98 | 1.58 |  | 0.37 | 0.87 | 0.64 | 1.18 |  |
|  |  | >3 | <0.001 | 2.80 | 2.26 | 3.46 |  | <0.001 | 4.06 | 3.28 | 5.04 |  |
| Number of persons/ household (1-5) | 2010 | >5 | <0.001 | 2.10 | 1.58 | 2.79 |  | <0.001 | 4.82 | 3.70 | 6.269 |  |
|  | 2019 | >5 | 0.26 | 2.03 | 0.52 | 8.04 | 0.06 | 0.12 | 4.08 | 0.59 | 28.15 | 0.01 |
| Familys´ disposable  income  (>highest quartile) | 2010 | <lowest quintile | <0.001 | 2.52 | 1.93 | 3.30 |  | <0.001 | 11.76 | 7.85 | 17.64 |  |
|  |  | In between | <0.01 | 1.45 | 1.15 | 1.83 |  | <0.001 | 3.26 | 2.19 | 4.86 |  |
|  | 2019 | <lowest quintile | <0.001 | 2.76 | 2.18 | 3.49 | 0.85 | <0.001 | 8.74 | 6.34 | 12.05 | 0.15 |
|  |  | In between | <0.001 | 1.47 | 1.20 | 1.81 |  | <0.001 | 3.22 | 2.36 | 4.37 |  |
| Financial  assistance (No) | 2010 | Yes | <0.001 | 4.15 | 3.11 | 5.54 |  | <0.001 | 16.39 | 12.61 | 21.29 |  |
|  | 2019 | Yes | <0.001 | 4.10 | 2.77 | 6.06 | 0.91 | <0.001 | 8.34 | 5.83 | 11.91 | <0.01 |
| Housing allowance (No) | 2010 | Yes | <0.001 | 2.83 | 2.28 | 3.51 |  | <0.001 | 8.49 | 6.80 | 10.60 |  |
|  | 2019 | Yes | <0.001 | 3.01 | 2.49 | 3.62 | 0.67 | <0.001 | 6.76 | 5.59 | 8.17 | 0.13 |
| Form of housing (Owning house) | 2010 | Renting | <0.001 | 2.66 | 2.20 | 3.23 |  | <0.001 | 9.56 | 7.54 | 12.12 |  |
|  |  | Owning apartment | 0.11 | 1.48 | 0.92 | 2.38 |  | <0.001 | 2.89 | 1.63 | 5.13 |  |
|  | 2019 | Renting | <0.001 | 2.61 | 2.11 | 3.23 |  | <0.001 | 6.37 | 4.14 | 9.80 | 0.01 |
|  |  | Owning apartment | <0.01 | 2.13 | 1.34 | 3.37 | 0.22 | 0.11 | 1.61 | 0.89 | 2.90 |  |
| Family living in in urban/ rural area (rural) | 2010 | urban | <0.01 | 1.41 | 1.09 | 1.83 |  | <0.001 | 2.99 | 2.00 | 4.46 |  |
|  | 2019 | urban | <0.001 | 1.92 | 1.48 | 2.49 | 0.10 | <0.001 | 3.06 | 2.15 | 4.35 | 0.93 |
