# Supplementary material for: Persistent oral health inequality in children—repeated cross-sectional studies in 2010 and 2019
Source: BMC Public Health. 2024 Dec 18;24:3528. doi: 10.1186/s12889-024-20905-y (PMC11658173; doi:10.1186/s12889-024-20905-y)
Supplement: Supplementary file 4 — Supplementary Material 4. [file 12889_2024_20905_MOESM4_ESM.docx]

Appendix 4. The association between caries and socioeconomic variables at residential area level year 2010 respectively 2019 for moderate and severe caries and the interaction over time.

|  |  |  | Comparison dmft 1-3 against dmft 0 | | | | | Comparison dmft >3 against dmft 0 | | | | | | | | | |
| --- | --- | --- | --- | --- | --- | --- | --- | --- | --- | --- | --- | --- | --- | --- | --- | --- | --- |
| Variable (ref) | **Year** | **Category** | ***p*-value** | **OR** | **CI**  **Lower** | **CI**  **Upper** | ***p*-interaction** | ***p*-value** | | **OR** | **CI**  **Lower** | | **CI**  **Upper** | | ***p*-interaction** | | |
| Ethnicity (Proportion born in Sweden) | **2010** | **Proportion born outside Sweden** | <0.001 | 1.04 | 1.04 | 1.05 |  | <0.001 | 1.09 | | | 1.08 | | 1.10 | |  |  |
|  | **2019** |  | <0.001 | 1.03 | 1.03 | 1.04 | 0.02 | <0.001 | 1.06 | | | 1.05 | | 1.06 | | <0.001 |  |
| Migration background (Proportion with native migration background) | **2010** | **Proportion with foreign background**^4^ | <0.001 | 1.03 | 1.03 | 1.04 |  | <0.001 | 1.07 | | | 1.06 | | 1.07 | |  |  |
|  | **2019** |  | <0.001 | 1.02 | 1.02 | 1.03 | 0.03 | <0.001 | 1.04 | | | 1.04 | | 1.04 | | <0.001 |  |
| Maternal age when first child was born | **2010** | **Average age of mothers at the birth of their first child** | <0.001 | 0.90 | 0.87 | 0.92 |  | <0.001 | 0.80 | | | 0.77 | | 0.83 | |  |  |
|  | **2019** |  | <0.001 | 0.94 | 0.92 | 0.97 | 0.02 | <0.001 | 0.82 | | | 0.79 | | 0.85 | | 0.35 |  |
| Paternal age when their first child was born | **2010** | **Average age of fathers at the birth of their first child** | <0.01 | 0.96 | 0.93 | 0.98 |  | <0.001 | 0.86 | | | 0.83 | | 0.90 | |  |  |
|  | **2019** |  | 0.76 | 1.00 | 0.97 | 1.02 | 0.03 | 0.001 | 0.96 | | | 0.93 | | 0.98 | | <0.001 |  |
| Employment status | **2010** | **Proportion without employment** | <0.001 | 1.06 | 1.05 | 1.07 |  | <0.001 | 1.12 | | | 1.10 | | 1.13 | |  |  |
|  | **2019** |  | <0.001 | 1.04 | 1.03 | 1.05 | 0.06 | <0.001 | 1.08 | | | 1.07 | | 1.10 | | <0.01 |  |
| Educational level (Proportion with higher education) | **2010** | **Proportion with highest education elementary school** | <0.001 | 1.05 | 1.03 | 1.07 |  | <0.001 | 1.10 | | | 1.09 | | 1.12 | |  |  |
|  |  | **Proportion with highest education highschool** | 0.19 | 1.01 | 1.00 | 1.02 |  | <0.001 | 0.97 | | | 0.96 | | 0.99 | |  |  |
|  |  | **Else** | <0.001 | 1.11 | 1.06 | 1.16 |  | <0.001 | 1.13 | | | 1.08 | | 1.18 | |  |  |
|  | **2019** | **Proportion with highest education elementary school** | <0.001 | 1.04 | 1.03 | 1.05 | 0.25 | <0.001 | 1.07 | | | 1.06 | | 1.08 | | <0.01 |  |
|  |  | **Proportion with highest education highschool** | 0.80 | 1.00 | 0.99 | 1.01 | 0.21 | 0.56 | 1.00 | | | 0.99 | | 1.01 | | <0.001 |  |
|  |  | **Else** | <0.001 | 1.08 | 1.05 | 1.12 | 0.41 | <0.001 | 1.14 | | | 1.10 | | 1.17 | | 0.46 |  |
| Family type  (not singel) | **2010** | **Proportion of single mothers** | <0.001 | 1.12 | 1.09 | 1.15 |  | <0.001 | 1.23 | | | 1.20 | | 1.27 | |  |  |
|  | **2019** |  | <0.001 | 1.10 | 1.07 | 1.12 | 0.64 | <0.001 | 1.17 | | | 1.15 | | 1.20 | | <0.01 |  |
| Family type  (not singel) | **2010** | **Proportion of single fathers** | 0.57 | 0.98 | 0.90 | 1.06 |  | 0.01 | 0.86 | | | 0.77 | | 0.96 | |  |  |
|  | **2019** |  | 0.01 | 0.91 | 0.84 | 0.98 | 0.22 | <0.001 | 0.77 | | | 0.71 | | 0.84 | | 0.14 |  |
| Number of children in the household | **2010** | **Average number of children/ household** | <0.001 | 0.53 | 0.39 | 0.73 |  | <0.01 | 0.53 | | | 0.37 | | 0.76 | |  |  |
|  | **2019** |  | 0.11 | 0.81 | 0.63 | 1.05 | 0.04 | <0.01 | 0.61 | | | 0.46 | | 0.82 | | 0.56 |  |
| Number of persons/ household | **2010** | **Average number of persons/ household** | <0.001 | 0.55 | 0.45 | 0.68 |  | <0.001 | 0.41 | | | 0.32 | | 0.52 | |  |  |
|  | **2019** |  | <0.001 | 0.69 | 0.58 | 0.83 | 0.12 | <0.001 | 0.44 | | | 0.36 | | 0.54 | | 0.62 |  |
| Disposable income per consumption unit/ household | **2010** | **Median disposable income per consumption unit/ household (divided by 10<0.001)** | <0.001 | 0.32 | 0.25 | 0.39 |  | <0.001 | 0.07 | | | 0.05 | | 0.09 | |  |  |
|  | **2019** |  | <0.001 | 0.41 | 0.35 | 0.48 | 0.04 | <0.001 | 0.16 | | | 0.13 | | 0.19 | | <0.001 |  |
| High-income households | **2010** | **Proportion of high-income households** | <0.001 | 0.90 | 0.88 | 0.92 |  | <0.001 | 0.78 | | | 0.76 | | 0.80 | |  |  |
|  | **2019** |  | <0.001 | 0.91 | 0.90 | 0.93 | 0.34 | <0.001 | 0.83 | | | 0.81 | | 0.84 | | <0.001 |  |
| Financial assistance | **2010** | **Proportion of households with financial assistance** | <0.001 | 1.07 | 1.06 | 1.09 |  | <0.001 | 1.13 | | | 1.12 | | 1.14 | |  |  |
|  | **2019** |  | <0.001 | 1.06 | 1.05 | 1.07 | 0.15 | <0.001 | 1.10 | | | 1.09 | | 1.11 | | <0.01 |  |
| Housing allowance | **2010** | **Proportion of households with housing allowance** | <0.001 | 1.06 | 1.05 | 1.07 |  | <0.001 | 1.12 | | | 1.11 | | 1.13 | |  |  |
|  | **2019** |  | <0.001 | 1.06 | 1.05 | 1.07 | 0.61 | <0.001 | 1.10 | | | 1.09 | | 1.11 | | <0.001 |  |
| Form of housing (Proportion owning house) | **2010** | **Proportions housing in renting** | <0.001 | 1.01 | 1.01 | 1.02 |  | <0.001 | 1.03 | | | 1.03 | | 1.04 | |  |  |
|  |  | **Proportion owning appartment in appartment building** | 0.51 | 1.00 | 0.99 | 1.00 |  | 0.21 | 1.01 | | | 1.00 | | 1.01 | |  |  |
|  |  | **Else** | 0.73 | 1.00 | 0.98 | 1.01 |  | <0.01 | 1.03 | | | 1.01 | | 1.04 | |  |  |
|  | **2019** | **Proportions housing in renting** | <0.001 | 1.01 | 1.01 | 1.02 | 0.65 | <0.001 | 1.03 | | | 1.02 | | 1.03 | | 0.65 |  |
|  |  | **Proportion owning appartment in appartment building** | 0.07 | 1.00 | 1.00 | 1.01 | 0.10 | 0.04 | 1.01 | | | 1.00 | | 1.01 | | 0.10 |  |
|  |  | **Else** | 0.49 | 1.01 | 0.99 | 1.02 | 0.50 | 0.08 | 1.01 | | | 1.00 | | 1.03 | | 0.50 |  |
| Urban/ rural area (rural area) | **2010** | **Proportion of households in urban area** | <0.01 | 1.00 | 1.00 | 1.01 |  | <0.001 | 1.01 | | | 1.01 | | 1.02 | |  |  |
|  | **2019** |  | <0.001 | 1.01 | 1.00 | 1.01 | 0.07 | <0.001 | 1.01 | | | 1.01 | | 1.02 | | 0.91 |  |
| Residential area CNI (<1) | **2010** | **>1** | <0.001 | 1.98 | 1.65 | 2.38 |  | <0.001 | 5.99 | | | 4.55 | | 7.87 | |  |  |
|  | **2019** | **>1** | <0.001 | 1.88 | 1.60 | 2.21 | 0.68 | <0.001 | 4.72 | | | 3.79 | | 5.86 | | 0.18 |  |
